# Supplementary material for: The IL-1β, IL-6, and TNF cytokine triad is associated with post-acute sequelae of COVID-19
Source: Cell Rep Med. 2022 Jun 21;3(6):100663. doi: 10.1016/j.xcrm.2022.100663 (PMC9214726; doi:10.1016/j.xcrm.2022.100663)
Supplement: Document S1. Tables S1 and S2 [file mmc1.pdf]

Cell Reports Medicine, Volume *3*

Supplemental information

The IL-1β, IL-6, and TNF cytokine triad

is associated with post-acute sequelae of COVID-19

Christoph Schultheiß, Edith Willscher, Lisa Paschold, Cornelia Gottschick, Bianca Klee, Svenja-Sibylla Henkes, Lidia Bosurgi, Jochen Dutzmann, Daniel Sedding, Thomas Frese, Matthias Girndt, Jessica I. Höll, Michael Gekle, Rafael Mikolajczyk, and Mascha Binder

Table S1: Characteristics of participants in the DigiHero „COVID-19“ module, related to Figure 3 – validation cohort

	all participants	participants with confirmed prior COVID-19	Participants without prior COVID-19
Nb of participants	300	240	60
Sex			
Female	171 (57%)	143 (59.6%)	28 (46.7%)
Male	129 (43%)	97 (40.4%)	32 (53.3%)
Age (years)			
Median age	44	44.7	38
Range	14-87	14-86	14-87
Timing of COVID-19 infections			
Wave 1 (March 2020 -June 2020)		6 (2.5%)	
Wave 2 (July 2020 - Feb 2021)		153 (63.8%)	
Wave 3 (Mar 2021 - June 2021)		63 (26.3%)	
Wave 4 (from July 2021)		17 (7%)	
Not evaluated		1 (0.4%)	
Self rated acute infection severity			
asymptomatic		15 (6.3%)	
mild to moderate		208 (86.7%)	
severe		16 (6.6%)	
Not evaluated		1 (0.4%)	
Hospitalization		8 (3%)	
Intensive care unit		0	
Duration of symptoms			
not evaluated		18 (7.5%)	
0-4 weeks		75 (31.3%)	
4-12 weeks		29 (12%)	
>12 weeks		118 (49.2%)	
Nb of SARS-COV-2 vaccinations			
0		30 (12.5%)	7 (11.7%)
1		129 (53.75%)	6 (10%)
2		81 (33.75%)	47 (78.3%)
Type of SARS-CoV-2 vaccine			
not evaluated		13 (6.2%)	5 (9.4%)
mRNA vaccine*		122 (58.1%)	4 (7.55%)
mRNA vaccine + mRNA vaccine		53 (25.2%)	31 (58.5%)
vector vaccine*		7 (3.3%)	2 (3.8%)
vector vaccine + vector vaccine		5 (2.4%)	4 (7.55%)
vector vaccine + mRNA vaccine		10 (4.8%)	7 (13.2%)

* mRNA vaccines: Comirnaty (BioNTech / Pfizer) or Spikevax (Moderna Biotech),
vector vaccines: Vaxzevria (AstraZeneca) or Ad26.COV2-S (Johnson&Johnson)

Table S2: Duration of symptoms, related to Figure 3 – validation cohort

	< 4w		4-12w		>12w		ongoing	
Nb of participants with symptoms	217		143		97		65	
Fever	138	63.6%	7	4.9%	3	3%	1	1.5%
Lymph node swelling	56	25.8%	10	7%	5	5.2%	4	6.2%
Loss of taste/smell	167	77%	81	56.6%	37	38.1%	26	40%
Body aches	186	85.7%	59	41.3%	36	37.1%	29	44.6%
Fatigue	202	93.1%	105	73.4%	68	70.1%	49	75.4%
Sleeping disturbances	113	52.1%	61	42.66%	47	48.45%	38	58.5%
Lack of concentration	119	54.8%	81	56.6%	50	51.55%	40	61.5%
Anxiety	63	29%	34	23.8%	25	25.8%	21	32.3%
Depression	36	16.9%	34	23.8%	25	25.8%	22	33.8%
Headache	181	83.4%	54	37.8%	27	27.8%	20	30.8%
Coryza	168	77.4%	25	17.5%	16	16.5%	14	21.5%
Conjunctivitis	19	8.7%	5	3.5%	3	3.1%	2	3.1%
Otitis	42	19.3%	14	9.8%	10	10.3%	9	13.8%
Dyspnea	148	68.2%	87	60.8%	59	60.8%	44	67.7%
Sore throat	141	65%	24	16.8%	10	10.3%	9	13.8%
Cough	166	76.5%	51	35.7%	23	23.7%	17	26.2%
Angina	125	57.6%	54	37.8%	37	38.1%	29	44.6%
Heart complaints	46	21.2%	29	20.3%	25	25.8%	21	32.3%
Dizziness	109	50.2%	48	33.6%	29	29.9%	26	40%
Abdominal pain	48	22.1%	15	10.5%	11	11.3%	9	13.8%
Gastrointestinal compl	68	31.3%	23	16%	17	17.5%	15	23.1%
Nausea	41	18.9%	11	7.7%	8	8.3%	8	12.3%

w, weeks
