# Supplementary material for: The IL-1β, IL-6, and TNF cytokine triad is associated with post-acute sequelae of COVID-19
Source: Cell Rep Med. 2022 Jun 21;3(6):100663. doi: 10.1016/j.xcrm.2022.100663 (PMC9214726; doi:10.1016/j.xcrm.2022.100663)
Supplement: Document S2. Article plus supplemental information [file mmc2.pdf]

Cell Reports Medicine

Article

The IL-1β, IL-6, and TNF cytokine triad is associated with post-acute sequelae of COVID-19

Graphical abstract

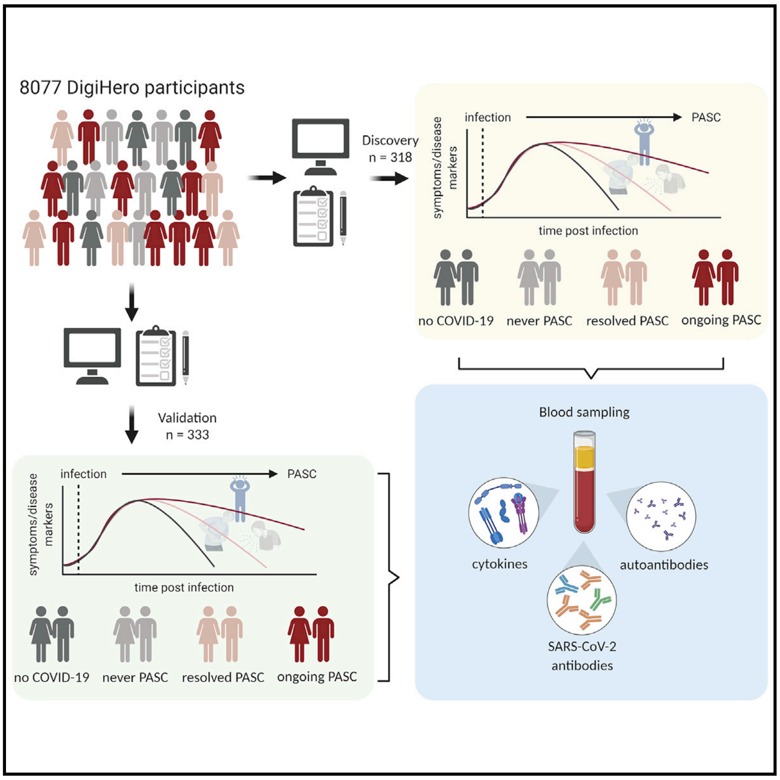

Authors

Christoph Schultheiß, Edith Willscher, Lisa Paschold, ..., Michael Gekle, Rafael Mikolajczyk, Mascha Binder

Correspondence

mascha.binder@uk-halle.de

In brief

By using a digital epidemiology approach including blood sampling, Schultheiβ et al. report post-acute sequelae persisting up to 24 months in 60% of patients after mild COVID-19. Plasma cytokine analysis reveals association with elevated levels of IL-1β, IL-6, and TNF potentially secreted from overactivated monocytes/macrophages.

Highlights

- We report a post-COVID-19 digital epidemiology study with biomarker analysis (n = 651)
- PASC persists in 60% of participants up to 24 months after mild COVID-19
- PASC is associated with high IL-1β, IL-6, and TNF levels but not autoantibodies
- Overactivated monocytes/macrophages are likely the source of cytokine production

Schultheiß et al., 2022, Cell Reports Medicine 3, 100663
June 21, 2022 © 2022 The Author(s).
https://doi.org/10.1016/j.xcrm.2022.100663

CellPress

Cell Reports Medicine

CellPress
OPEN ACCESS

Article

The IL-1β, IL-6, and TNF cytokine triad is associated with post-acute sequelae of COVID-19

Christoph Schultheiß,[1] Edith Willscher,[1] Lisa Paschold,[1] Cornelia Gottschick,[2] Bianca Klee,[2] Svenja-Sibylla Henkes,[1] Lidia Bosurgi,[3,4] Jochen Dutzmann,[5] Daniel Sedding,[5] Thomas Frese,[6] Matthias Girndt,[7] Jessica I. Höll,[8] Michael Gekle,[9] Rafael Mikolajczyk,[2] and Mascha Binder[1,10,11,*]

[1]Department of Internal Medicine IV, Oncology/Hematology, Martin-Luther-University Halle-Wittenberg, Ernst-Grube-Str. 40, 06120 Halle (Saale), Germany

[2]Institute for Medical Epidemiology, Biometrics and Informatics (IMEBI), Interdisciplinary Center for Health Sciences, Medical School of the Martin-Luther University Halle-Wittenberg, Magdeburger Strasse 8, 06097 Halle (Saale), Germany

[3]I. Department of Medicine, University Medical Center Hamburg-Eppendorf, Martinistrasse 52, 20251 Hamburg, Germany

[4]Protozoa Immunology, Bernhard Nocht Institute for Tropical Medicine, Bernhard Nocht Strasse 74, 20359 Hamburg, Germany

[5]Mid-German Heart Center, Department of Cardiology and Intensive Care Medicine, University Hospital, Martin-Luther-University Halle--Wittenberg, Ernst-Grube-Str. 40, 06120 Halle (Saale), Germany

[6]Institute of General Practice and Family Medicine, Martin-Luther-University Halle-Wittenberg, Magdeburger Str. 8, 06112 Halle (Saale), Germany

[7]Department of Internal Medicine II, Martin-Luther-University Halle-Wittenberg, Ernst-Grube-Str. 40, 06120 Halle (Saale), Germany

[8]Pediatric Hematology and Oncology, Martin-Luther-University Halle-Wittenberg, Ernst-Grube-Str. 40, 06120 Halle (Saale), Germany

[9]Julius Bernstein-Institute of Physiology, Faculty of Medicine, Martin-Luther-University Halle-Wittenberg, Magdeburger Str. 6, 06110 Halle (Saale), Germany

[10]Twitter: @lab_binder

[11]Lead contact

*Correspondence: mascha.binder@uk-halle.de

https://doi.org/10.1016/j.xcrm.2022.100663

SUMMARY

Post-acute sequelae of COVID-19 (PASC) is emerging as global problem with unknown molecular drivers. Using a digital epidemiology approach, we recruited 8,077 individuals to the cohort study for digital health research in Germany (DigiHero) to respond to a basic questionnaire followed by a PASC-focused survey and blood sampling. We report the first 318 participants, the majority thereof after mild infections. Of those, 67.8% report PASC, predominantly consisting of fatigue, dyspnea, and concentration deficit, which persists in 60% over the mean 8-month follow-up period and resolves independently of post-infection vaccination. PASC is not associated with autoantibodies, but with elevated IL-1β, IL-6, and TNF plasma levels, which we confirm in a validation cohort with 333 additional participants and a longer time from infection of 10 months. Blood profiling and single-cell data from early infection suggest the induction of these cytokines in COVID-19 lung pro-inflammatory macrophages creating a self-sustaining feedback loop.

INTRODUCTION

Severe acute respiratory syndrome coronavirus 2 (SARS-CoV-2) is a new virus causing coronavirus disease 2019 (COVID-19) that has led to a health crisis of global scale.[1,2] COVID-19 is now recognized as a multi-organ disease with considerable mortality in risk groups.[3–5] With a growing population of recovering patients, it became clear that in 32% to 87% of patients (including those with mild acute disease), health impairments persist beyond the acute phase of infection.[6–12] The most common definition of such post-acute sequelae of COVID-19 (PASC) is persistence of symptoms beyond 4 weeks.[6,7] The clinical spectrum of PASC includes fatigue and exercise intolerance, brain fog, shortness of breath, joint pain, fever, sleep and anxiety disorders, as well as gastrointestinal symptoms and palpitations.[6–8,11] Symptoms may persist for months and their severity can range from mild to debilitating. The immense numbers of COVID-19 survivors with post-infection disability that prevents these individuals from returning to normal active life added another layer in this health crisis beyond the threat of exhausting intensive care unit capacities. Given more than 400 million SARS-CoV-2 infections counted globally in early March 2022 by the World Health Organization, the impact of PASC will likely be profound.

The pathophysiology of PASC after mild or moderate infection is still largely unexplored,[6,8] may differ from PASC after intensive care treatment with respect to affected population groups and symptoms,[12] and targeted treatment approaches are lacking. While some of the delayed symptoms may be a consequence of virus-induced tissue injury affecting multiple organs,[13,14] another potential trigger has been proposed to result from persistent SARS-CoV-2 reservoirs. This hypothesis has been fueled by the observation that some infected patients do not rapidly clear the virus,[15–18] which is in line with the observation that post-acute viral persistence is a relatively common feature of RNA viruses

This is an open access article under the CC BY license (http://creativecommons.org/licenses/by/4.0/).

(e.g., Ebola and hepatitis C virus) and has also been discussed in the context of chronic symptoms or reactivated disease.[19] Yet, direct evidence pointing to a role of such potential reservoirs in PASC, as well as on the effects of their eradication, e.g., via post-infection vaccination, is currently lacking.[20] Another potential biological correlate of PASC may be autoimmune tissue damage. Already in the early phases of the pandemic, it became obvious that the SARS-CoV-2 virus shifts adaptive immunity toward autoreactivity.[21,22] There is now a large body of evidence that diverse autoantibody classes are produced in acute COVID-19 as well as post-COVID-19 multisystem inflammatory syndrome in children.[22–29] Moreover, many reports suggest that patients may experience *de novo* or worsening of preexisting autoimmune conditions, such as autoimmune cytopenias, Guillain-Barré syndrome, or systemic lupus erythematosus.[30,31] It remains elusive, however, if autoantibodies represent an inflammatory epiphenomenon or pathophysiologically contribute to PASC.[32,33]

Quickly closing the knowledge gap on PASC pathophysiology is one of the current global priorities. Here, we show how the combination of digital epidemiology with selective biobanking can rapidly generate hints toward disease mechanisms. Using this approach, we rapidly identified and recruited a large cohort allowing dedicated analyses of biomaterial in a subsample of previously infected participants with or without PASC. Our analysis provides evidence for a long-lasting cytokine signature consisting of elevated levels of interleukin (IL)-1β, IL-6, and tumor necrosis factor (TNF) that potentially underlies many of the clinical symptoms of PASC and that may derive from the macrophage compartment.

RESULTS

Characteristics of participants in the DigiHero COVID-19 module

As a discovery cohort, we included 318 participants from the DigiHero study who had been recruited until October 9, 2021, and who had indicated prior COVID-19 in their household. A total of 258 individuals thereof had COVID-19 themselves (as confirmed by a positive PCR or antigen test) and 36 were presumably uninfected household members (no symptoms, no positive PCR or antigen test, Figure 1A). Twenty-four individuals with suspected infection due to symptoms were excluded from the analysis due to lack of confirmed infection. Basic characteristics of the cohort are summarized in Table 1. More than 76.7% of the previously infected participants had COVID-19 or asymptomatic SARS-CoV-2 infections in Germany's second wave. More than 80% of acute infections were rated mild to moderate by the participants. Median time from positive PCR or antigen test to participation in the module was 8 months (Figure 1B). More than 80% of participants had received at least one dose of a COVID-19 vaccine.

SARS-CoV-2 antibody results confirm infection/vaccination status information provided by participants

To assess validity of the provided information on infection and vaccination status, we performed ELISA testing for SARS-CoV-2 NCP and S1 antibodies (Figure 1C). These results confirmed prior evidence that patients vaccinated after natural infection achieved the highest S1 antibody levels followed by vaccinated, but uninfected individuals.[34] The lowest S1 antibody levels were detected in individuals after natural infection without subsequent vaccination and the few individuals who were neither previously infected nor vaccinated. These data were well compatible with the information on infection/vaccination status provided by the participants except for two individuals who indicated no prior COVID-19 or vaccination despite elevated antibody levels. These two cases were excluded from further analyses.

COVID-19 symptoms and PASC in the DigiHero COVID-19 module

A total of 175 (67.8%) previously infected participants reported symptoms beyond 4 weeks from positive SARS-CoV-2 testing and were therefore considered to have PASC (Table 1). Distribution of acute COVID-19 and PASC symptoms are shown in Table 2. Of the participants with PASC who had not been hospitalized at the time of their acute infection, 20% showed symptoms only up to 12 weeks and about 60% had ongoing symptoms at the time of blood sampling (Figure 1D). About half of the participants with PASC reported at least one severe symptom beyond 4 weeks from positive SARS-CoV-2 testing (Figure 1E). Fatigue and dyspnea were among the most prevalent symptoms, with a considerable fraction of cases showing moderate to severe symptom load (Figure 1F). Women showed a higher percentage of PASC than men and the percentage of patients reporting PASC decreased with time (Figure 1G). While PASC incidence was independent of age (Figure 1H), participants with more severe acute infections more likely reported to be affected by PASC (Figure 1I).

Post-infection vaccination was not associated with resolution of PASC in the DigiHero cohort

Anecdotal reports suggested that SARS-CoV-2 vaccination may lead to resolution of PASC,[35] possibly via elimination of a cryptic viral reservoir by the induction of a refined immune response.[36] In our cohort, however, we found that the percentage of patients with ongoing PASC was similar in participants with post-infection vaccination and those without (Figure 1J). Median time point of post-infection vaccination was at month 6 (range 4–12) and the median time point from vaccination to study participation was 3 months (range 1–9).

Elevated autoantibody levels after COVID-19 were not associated with PASC

We performed autoantibody screens and correlated autoantibody positivity with clinical symptoms. We included rheumatoid factor (RF), antinuclear antibodies (ANAs), and anti-phospholipid antibodies (aPLs) in our assessment.[22] While the control cases without prior COVID-19 infection did not show positivity for any of the tested specificities, the percentage of participants with positive test results for one or more autoantibodies amounted to 20% of prior COVID-19 cases (Figures 2A and 2B). Interestingly, participants with earlier SARS-CoV-2 infections showed equal rates of autoantibody positivity compared with participants with later infections arguing against their short-lived nature (Figure 2B). Yet, neither positivity for these autoantibodies (Figure 2C) nor antibody levels (data not shown) correlated with PASC or sampling time point. Of note, in none of the participants,

CellPress
OPEN ACCESS

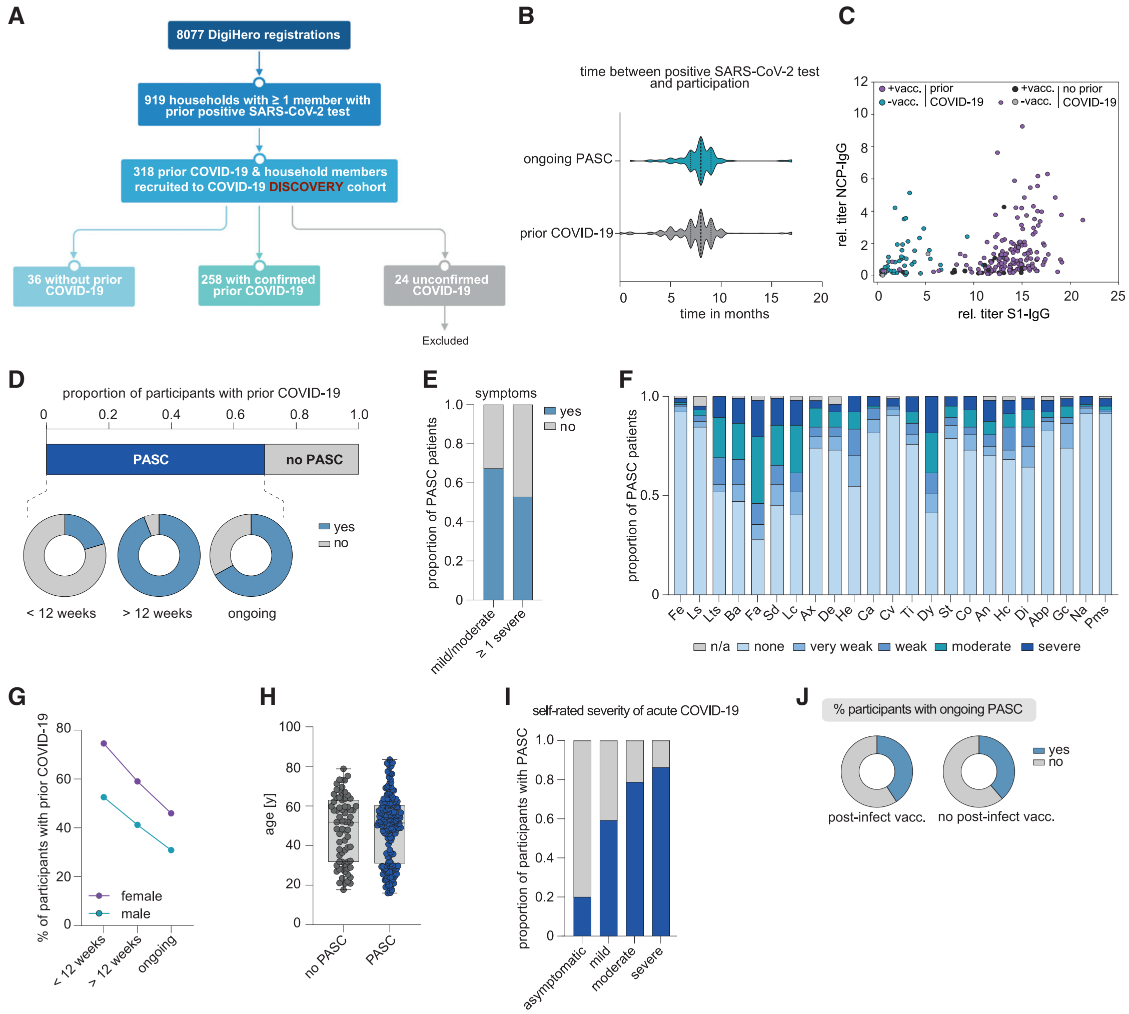

Figure 1. Clinical and epidemiological parameters of the DigiHero discovery cohort and patients with PASC
(A) Flow chart of the COVID-19 module of the DigiHero study.
(B) Median time from positive PCR or antigen test to participation in the module for the prior COVID-19 (n = 154) and ongoing PASC groups (n = 104).
(C) Plasma titer of antibodies directed against the S1 and NCP proteins of SARS-CoV-2 in individuals with or without SARS-CoV-2 vaccination (+vacc./−vacc.) and with or without prior COVID-19 from the DigiHero cohort.
(D) Proportion of DigiHero participants with self-reported PASC including duration of PASC symptoms after infection plus proportion of patients with ongoing symptoms at the time of blood sampling.
(E) Proportion of PASC patients with mild/moderate or at least one severe symptom.
(F) Severity of self-reported symptoms in PASC patients.
(G) Distribution of PASC duration between female and male study participants with prior COVID-19.
(H) Age distribution of DigiHero participants with or without PASC shown as box plot extending from the 25th to 75th percentiles. Median age is indicated as line. Bars represent range from smallest to highest value.
(I) Severity of acute COVID-19 in PASC patients. Abp, abdominal pain; An, angina; Ax, anxiety; Ba, body aches; Ca, coryza; Co, cough; Cv, conjunctivitis; De, depression; Di, dizziness; Dy, dyspnea; Fa, fatigue; Fe, fever; Gc, gastrointestinal complaints; He, headache; Hc, heart complaints; Lc, lack of concentration; Ls, lymph node swelling; Lts, loss of taste/smell; Na, nausea; Sai, self-reported severity of acute infection; SD, sleep disturbance; St, sore throat; Ti, tinnitus.
(J) Post-vaccination status of patients with ongoing PASC.

a new diagnosis of a *bona fide* autoimmune disorder was reported in the surveyed period. Two participants reported worsening of preexisting rheumatoid arthritis or psoriasis, respectively. However, these two cases tested negative for the autoantibodies included in our screening panel. Together, these data do not support an involvement of the tested autoantibody classes in the pathogenesis of PASC.

Participants with PASC show a pro-inflammatory blood cytokine profile

Next, we profiled plasma samples from participants without prior COVID-19, previously infected participants without PASC, and those with resolved or ongoing PASC for a panel of 21 cytokines that are deregulated in acute COVID-19.[37] Despite the long interval between acute infection and blood sampling of 8 months,

Table 1. Characteristics of participants in the DigiHero COVID-19 module

	All participants	Participants with confirmed prior COVID-19	Participants without prior COVID-19
No. of participants	318	258	36
Sex			
Female	192 (60.4%)	161 (62.4%)	22 (61.1%)
Male	126 (39.6%)	97 (37.6%)	14 (38.9%)
Age (years)			
Median age	51.3	51.2	50
Range	15–83	15–83	17–81
Timing of COVID-19 infections			
Wave 1 (March 2020–June 2020)		7 (2.7%)	
Wave 2 (July 2020–Feb 2021)		198 (76.7%)	
Wave 3 (Feb 2021–June 2021)		45 (17.4%)	
Wave 4 (from July 2021)		8 (3.1%)	
Self-rated acute infection severity			
Asymptomatic		15 (5.8%)	
Mild to moderate		221 (85.7%)	
Severe		22 (8.5%)	
Hospitalization		8 (3.1%)	
Intensive care unit		4 (1.5%)	
Duration of symptoms			
Not evaluated		12 (4.7%)	
0–4 weeks		71 (27.5%)	
4–12 weeks		30 (11.6%)	
>12 weeks		145 (56.2%)	
No. of SARS-COV-2 vaccinations			
0		62 (24%)	6 (16.7%)
1		137 (53.1%)	0
2		59 (22.9%)	30 (83.3%)
Type of SARS-CoV-2 vaccine		196	
Not evaluated		25 (12.8%)	1 (3.3%)
mRNA vaccine[a]		128 (65.3%)	
mRNA vaccine + mRNA vaccine		29 (14.8%)	24 (80%)
vector vaccine[a]		7 (3.6%)	
vector vaccine + vector vaccine		3 (1.5%)	1 (3.3%)
vector vaccine + mRNA vaccine		4 (2%)	4 (13.3%)

Related to Figure 1, discovery cohort.
[a]mRNA vaccines: Comirnaty (BioNTech/Pfizer) or Spikevax (Moderna Biotech), vector vaccines: Vaxzevria (AstraZeneca) or Ad26.COV2-S (Johnson & Johnson).

participants with prior COVID-19 showed patterns of systemic cytokine deregulation also found in acute COVID-19 or early recovery,[37] including TNF (TNF-α), LTA (TNF-β), IL-1β, IL-4, IL-6, IL-8, IL-13, and interferon (IFN)-α2 (Figure 2D). Of those, only IL-1β, IL-6, and TNF showed a significant correlation with PASC (Figure 2D). Interestingly, the levels of these three cytokines were positively correlated with each other in individual participants, indicating that they do not identify separate subsets of patients with PASC (Figure 2E). These data suggest that persistently elevated levels of IL-1β, IL-6, and TNF may be a hallmark of PASC.

Confirmation of PASC cytokine triad in validation cohort of 333 additional DigiHero participants

To verify these results, we included another 333 DigiHero participants with previous COVID-19 in their households as a validation cohort. Of these, 240 patients had prior COVID-19 themselves, 60 were uninfected household members, and 33 participants had suspected infection due to symptoms but were excluded from the analysis due to lack of confirmed infection (Figure 3A; Table S1). All participants of the validation cohort had answered the COVID-19 questionnaire and undergone blood sampling until February 18. Overall, the structure of this

CellPress
OPEN ACCESS

Table 2. Duration of symptoms

	<4 wk		4–12 wk		>12 wk		Ongoing	
No. of participants with symptoms	242		171		135		104	
Fever	159	65.7%	7	4.1%	7	5.2%	7	6.7%
Lymph node swelling	59	24.4%	16	9.4%	12	8.9%	11	10.6%
Loss of taste/smell	167	69.0%	91	53.2%	62	45.9%	50	48.1%
Body aches	205	84.7%	83	48.5%	61	45.2%	54	51.9%
Fatigue	229	94.6%	135	78.9%	91	67.4%	73	70.2%
Sleeping disturbances	135	55.8%	86	50.3%	63	46.7%	56	53.8%
Lack of concentration	135	55.8%	99	57.9%	73	54.1%	60	57.7%
Anxiety	84	34.7%	40	23.4%	28	20.7%	25	24%
Depression	62	25.6%	43	25.1%	31	23.0%	24	23.1%
Headache	203	83.9%	79	46.2%	57	42.2%	47	45.2%
Coryza	175	72.3%	23	13.5%	21	15.6%	19	18.3%
Conjunctivitis	21	8.7%	10	5.8%	10	7.4%	9	8.7%
Otitis	51	21.1%	31	18.1%	28	20.7%	25	24%
Dyspnea	141	58.3%	107	62.6%	73	54.1%	61	58.7%
Sore throat	152	62.8%	28	16.4%	24	17.8%	22	21.2%
Cough	175	72.3%	55	32.2%	31	23.0%	28	26.9%
Angina	106	43.8%	53	31.0%	36	26.7%	29	27.9%
Heart complaints	61	25.2%	47	27.5%	36	26.7%	31	29.8%
Dizziness	125	51.7%	48	28.1%	43	31.9%	36	34.6%
Abdominal pain	53	21.9%	19	11.1%	17	12.6%	16	15.4%
Gastrointestinal complications	94	38.8%	37	21.6%	30	22.2%	26	25%
Nausea	60	24.8%	14	8.2%	10	7.4%	9	8.7%

Related to Figure 1, discovery cohort.

validation cohort was comparable to the discovery cohort, as shown in Figures 3B–3J and Tables 1 and 2. Of note, the time between infection and blood sampling in the previously infected participants was longer, with 10 instead of 8 months.

Cytokine profiling of this validation cohort confirmed the data from the first cohort by showing substantially elevated levels of IL-1β, IL-6, and TNF in participants with ongoing PASC (Figure 4A). Given the higher prevalence of females with ongoing PASC, we next asked whether this is reflected in the PASC-associated cytokine levels. Combination of the discovery and validation cohorts allowed us to study this question with high statistical power. Interestingly, the PASC-associated cytokine elevations were independent of male or female sex (Figure 4B). Correlation of all profiled cytokines from the combined discovery and validation cohorts shows that IL-1β, IL-6, and TNF especially correlate with each other in ongoing PASC (Figure 4C). Notably, IL-6 also positively correlated with IL-12p70 and IFN-γ, although these cytokines showed no association with PASC in either of the analyzed cohorts.

Since 20% of participants in both cohorts report resolution of PASC between 4 and 12 weeks after infection (Figures 1D and 3D), we asked whether there are trends toward normalization of IL-1β, IL-6, and TNF levels with elapsed time after infection in individuals reporting ongoing symptoms. Correlation of post-infection sampling time points with plasma cytokine levels from both cohorts showed no clear time-dependent decrease of IL-1β, IL-6, and TNF plasma levels in participants with PASC (Figure 4D).

Single-cell analysis of lung and blood macrophages from COVID-19 patients with acute disease recapitulate cytokine profile found in PASC

While the triad of IL-1β, IL-6, and TNF was characteristic for cases with PASC, the evolution of these cytokines from acute infection to the PASC stage as well as their cellular compartment of origin remained elusive. Since the COVID-19 module of the DigiHero study did not include participants with active COVID-19, we were unable to extrapolate the dynamics of the three PASC-associated cytokines from early infection to post-acute disease phases. To close this gap, we performed plasma cytokine profiling on additional samples from our independent halle COVID cohort (HACO) cohort that recruited patients with active infections and in the early post-infection period (median sampling on day 41 after symptom onset, range 23–53)[37] and integrated them with all samples from the combined DigiHero discovery and validation cohorts. Twenty patients with mild to moderate acute COVID-19 (median sampling on day 16 after symptom onset, range 1–44) were included that matched the characteristics of the

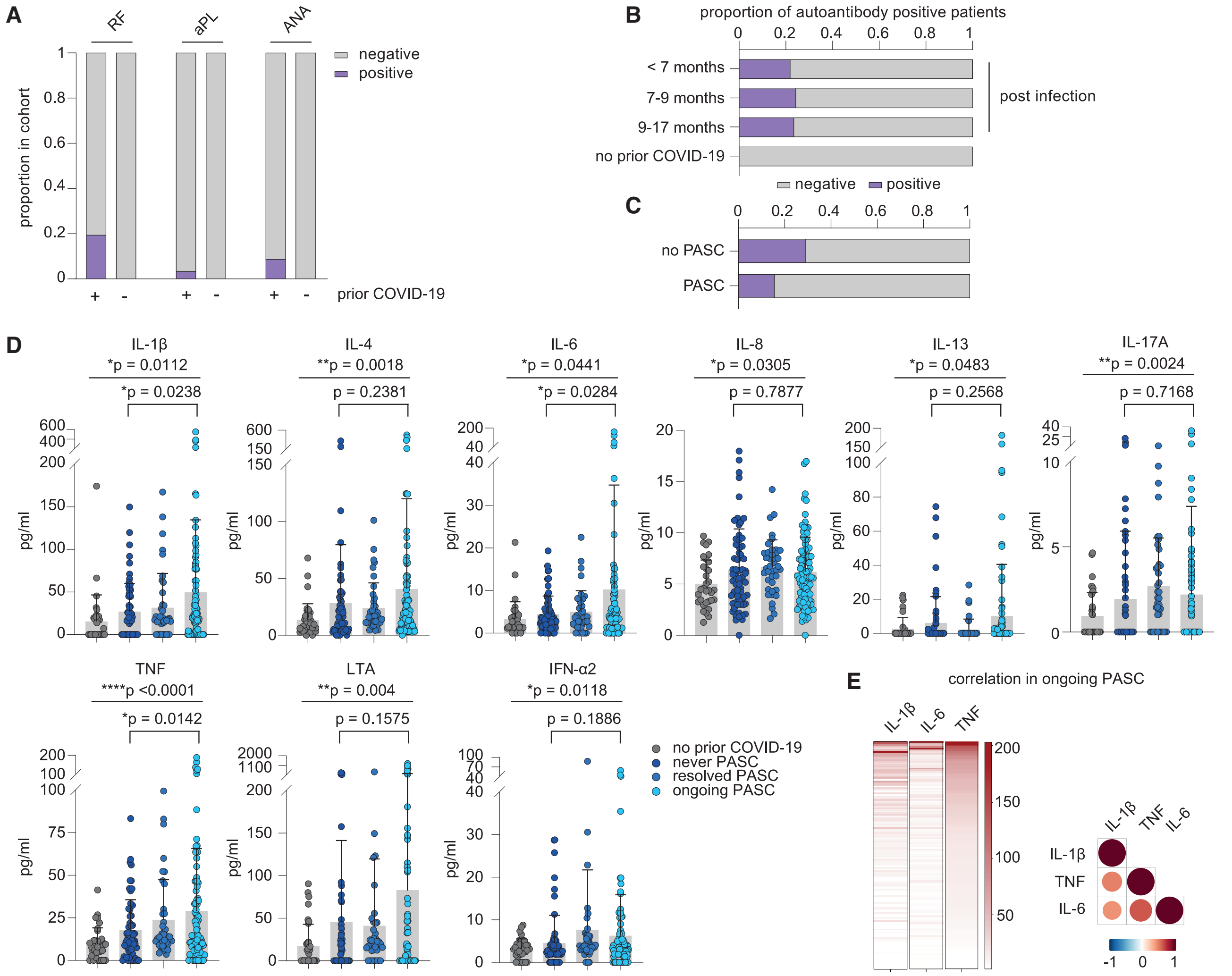

Figure 2. Serological profiling of plasma from patients of the discovery cohort with ongoing PASC, after resolved SARS-CoV-2 infection and after resolved PASC

(A) Proportion of participants with rheumatic factor (RF), and antinuclear (ANA) and phospholipid autoantibodies (aPL) dependent on COVID-19 history. n (prior COVID-19) = 201; n (no prior COVID-19) = 36.

(B) Seroprevalence of autoantibodies over time in COVID-19 patients. n (<7 months) = 41; n (7–9 months) = 152; n (>9 months) = 28.

(C) Seroprevalence of autoantibodies in patients with ongoing PASC and individuals after infection without developing PASC. n (PASC) = 96; n (no PASC) = 65.

(D) Mean plasma cytokine levels of participants who never reported PASC post-infection (n = 65), with ongoing PASC (n = 96), with resolved PASC (n = 41), and participants without prior COVID-19 (n = 36). Error bars indicate ± SD. Statistical analysis: Welch's ANOVA for comparison of all four groups and two-sided Welch corrected t test for comparison of never PASC versus ongoing PASC groups.

(E) Relation of IL-1β, IL-6, and TNF plasma levels in PASC patients displayed as heatmap (concentrations as pg/mL) and as correlation matrix.

DigiHero cohort. IL-1β and TNF levels were elevated in acute infection with concentrations exceeding those found in patients with bacterial pneumonia (Figure 5A). In line with the literature,[38] IL-6 levels were also elevated in acute COVID-19 as compared with individuals without infection but not as prominently as in the bacterial pneumonia samples. Despite the cross-sectional character of this analysis, the comparison of post-COVID-19 cytokine levels with those from individuals who never experienced COVID-19 suggests that all three cytokines remain elevated in post-acute disease phases but decrease in later post-infection phases.

Given that plasma cytokine levels decrease after SARS-CoV-2 infection while IL-1β, IL-6, and TNF remain stable in individuals with ongoing PASC, we next asked whether there are patterns in active COVID-19 that provide a mechanistical link to this observation. IL-1β, IL-6, and TNF are mainly secreted by monocytes and macrophages upon inflammatory stimuli.[46] The myeloid compartment and especially lung macrophages are strongly deregulated in COVID-19 patients as evidenced by previously published single-cell analysis on lung tissue, bronchoalveolar lavage fluid (BALF), and blood from patients with acute COVID-19.[39–42,44,47] To explore the potential cellular sources of

CellPress
OPEN ACCESS

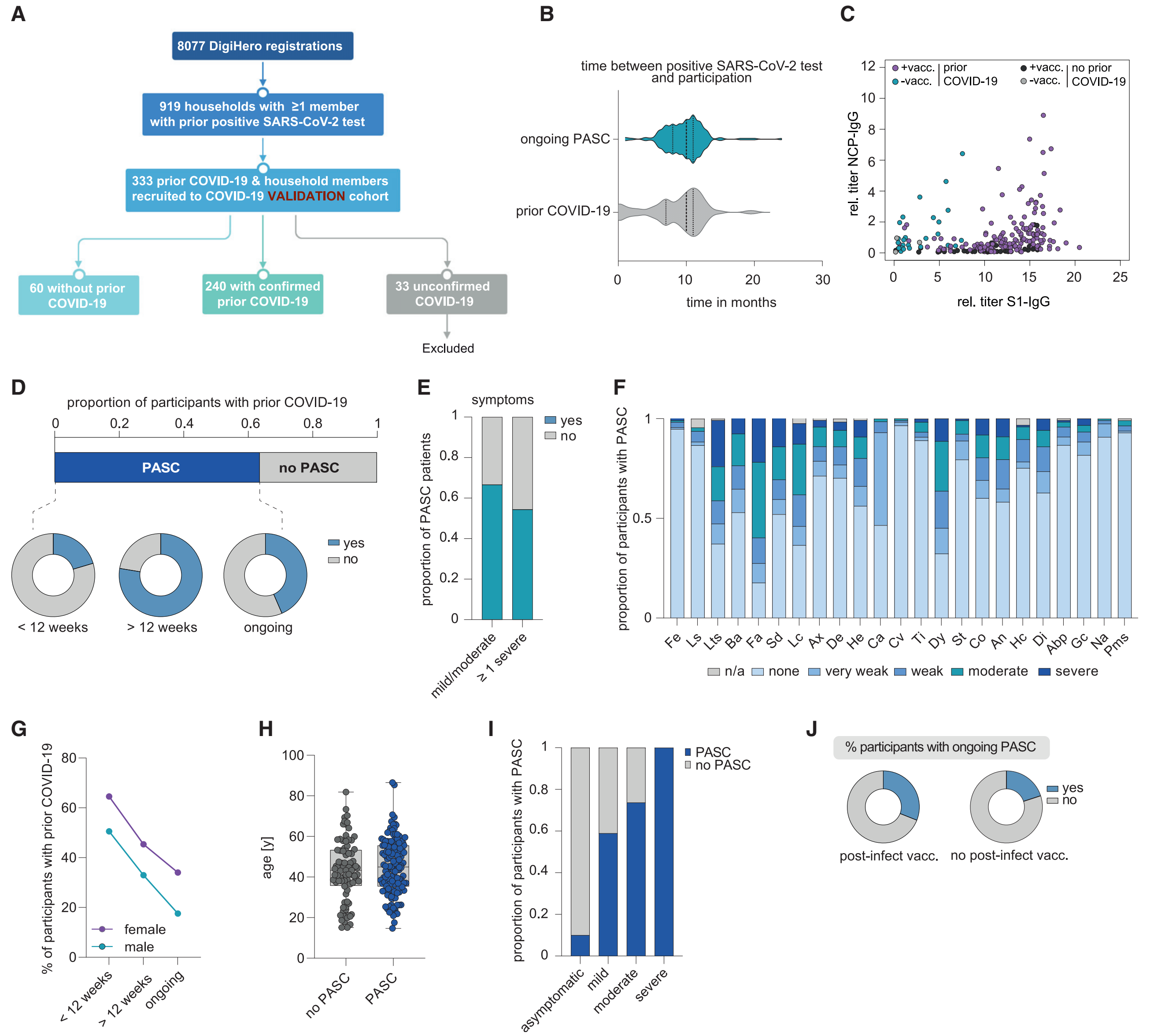

Figure 3. Clinical and epidemiological parameters of the DigiHero validation cohort and patients with PASC

(A) Flow chart of the COVID-19 module of the DigiHero study.

(B) Median time from positive PCR or antigen test to participation in the module for the prior COVID-19 (n = 87) and ongoing PASC groups (n = 153).

(C) Plasma titer of antibodies directed against the S1 and NCP proteins of SARS-CoV-2 in individuals with or without SARS-CoV-2 vaccination (+vacc./−vacc.) and with or without prior COVID-19 in the validation cohort.

(D) Proportion of DigiHero participants with self-reported PASC including duration of PASC symptoms after infection plus proportion of patients with ongoing symptoms at the time of blood sampling.

(E) Proportion of PASC patients with mild/moderate or at least one severe symptom.

(F) Severity of self-reported symptoms in PASC patients.

(G) Distribution of PASC duration between female and male study participants with prior COVID-19.

(H) Age distribution of DigiHero participants with or without PASC shown as box plot extending from the 25th to 75th percentiles. Median age is indicated as line. Bars represent range from smallest to highest value.

(I) Severity of acute COVID-19 in PASC patients.

Abp, abdominal pain; An, angina; Ax, anxiety; Ba, body aches; Ca, coryza; Co, cough; Cv, conjunctivitis; De, depression; Di, dizziness; Dy, dyspnea; Fa, fatigue; Fe, fever; Gc, gastrointestinal complaints; He, headache; Hc, heart complaints; Lc, lack of concentration; Ls, lymph node swelling; Lts, loss of taste/smell; Na, nausea; Sai, self-reported severity of acute infection; SD, sleep disturbance; St, sore throat; Ti, tinnitus.

(J) Post-vaccination status of patients with ongoing PASC.

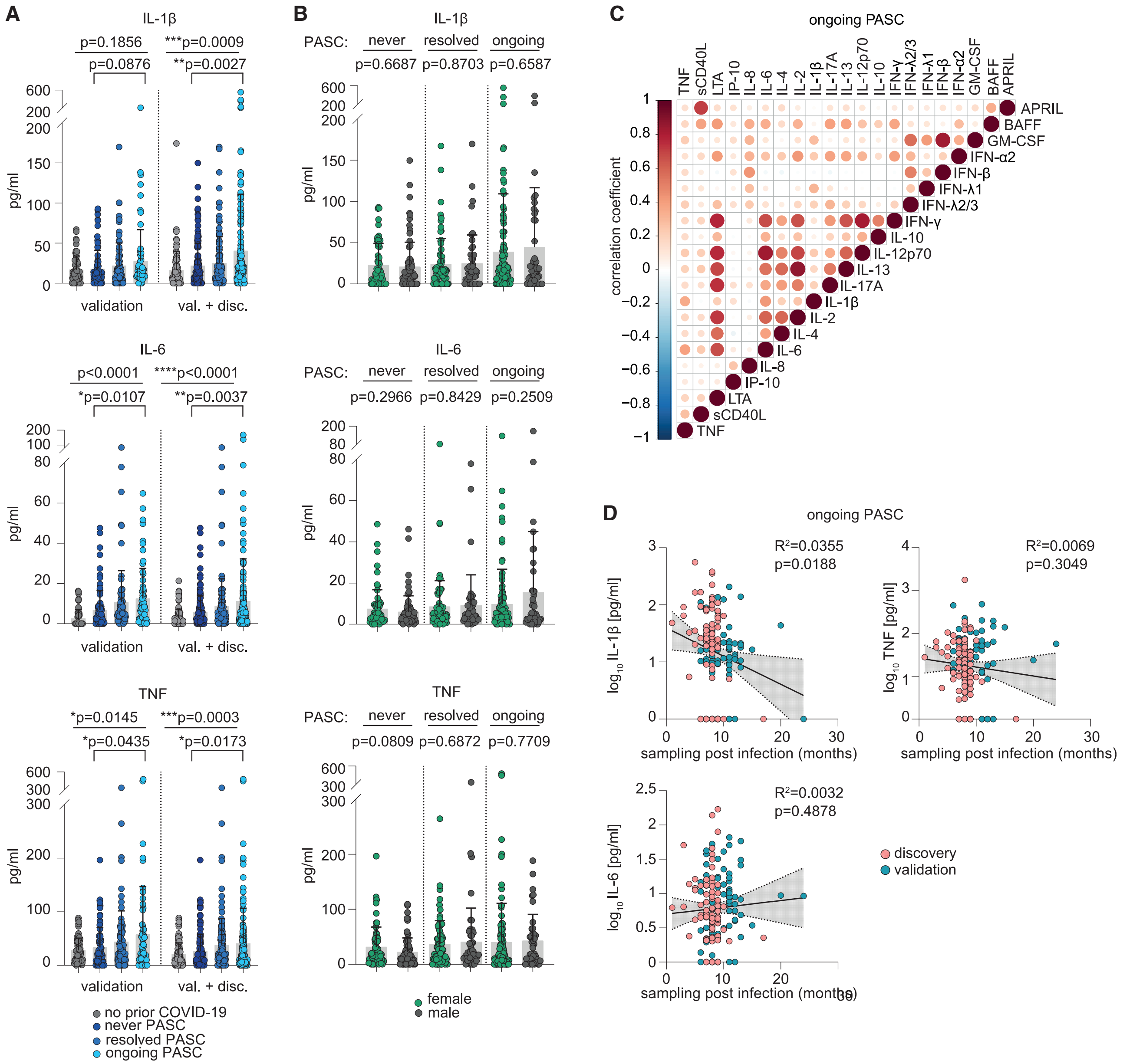

Figure 4. Serological profiling of plasma from patients of the validation cohort with ongoing PASC, after resolved SARS-CoV-2 infection and after resolved PASC

(A) Mean plasma cytokine levels of participants with prior COVID-19 from the validation cohort who never reported PASC (n = 86), with ongoing PASC (n = 89), or with resolved PASC (n = 65) and participants without prior COVID-19 (n = 60), as well as plasma cytokine levels in the combined discovery and validation cohorts: n (never COVID-19) = 96; n (no PASC) = 150; n (resolved PASC) = 106; n (ongoing PASC) = 185. Error bars indicate ± SD. Statistical analysis: Welch's ANOVA for comparison of all four groups and two-sided Welch corrected t test for comparison of never PASC versus ongoing PASC groups.

(B) Sex-dependent mean plasma cytokine levels in the never COVID-19 (71 females, 79 males), resolved PASC (77 females, 52 males), and ongoing PASC (116 females, 46 males) groups of the combined discovery and validation cohorts. Error bars indicate ± SD. Statistical analysis: two-sided Welch corrected t test.

(C) Correlation matrix of all cytokines in the combined ongoing PASC group (n = 185).

(D) Linear regression analysis of plasma cytokine levels and sampling time point post-infection in the combined ongoing PASC group (n = 185).

IL-1β, IL-6, and TNF in acute COVID-19, we analyzed previously published single-cell transcriptomics datasets from lung tissue, peripheral blood, and BALF of patients in acute infection.[39–45] This analysis confirmed that this triad of cytokines was specifically expressed by the myeloid/macrophage compartment in the lungs (Figure 5B) and in BALF-derived pro-inflammatory macrophages (Figure 5C). To identify gene expression patterns supporting the hypothesis of a self-stimulatory feedback loop for IL-1β, IL-6, and TNF, we next calculated response scores for these cytokines in the macrophage compartment based on

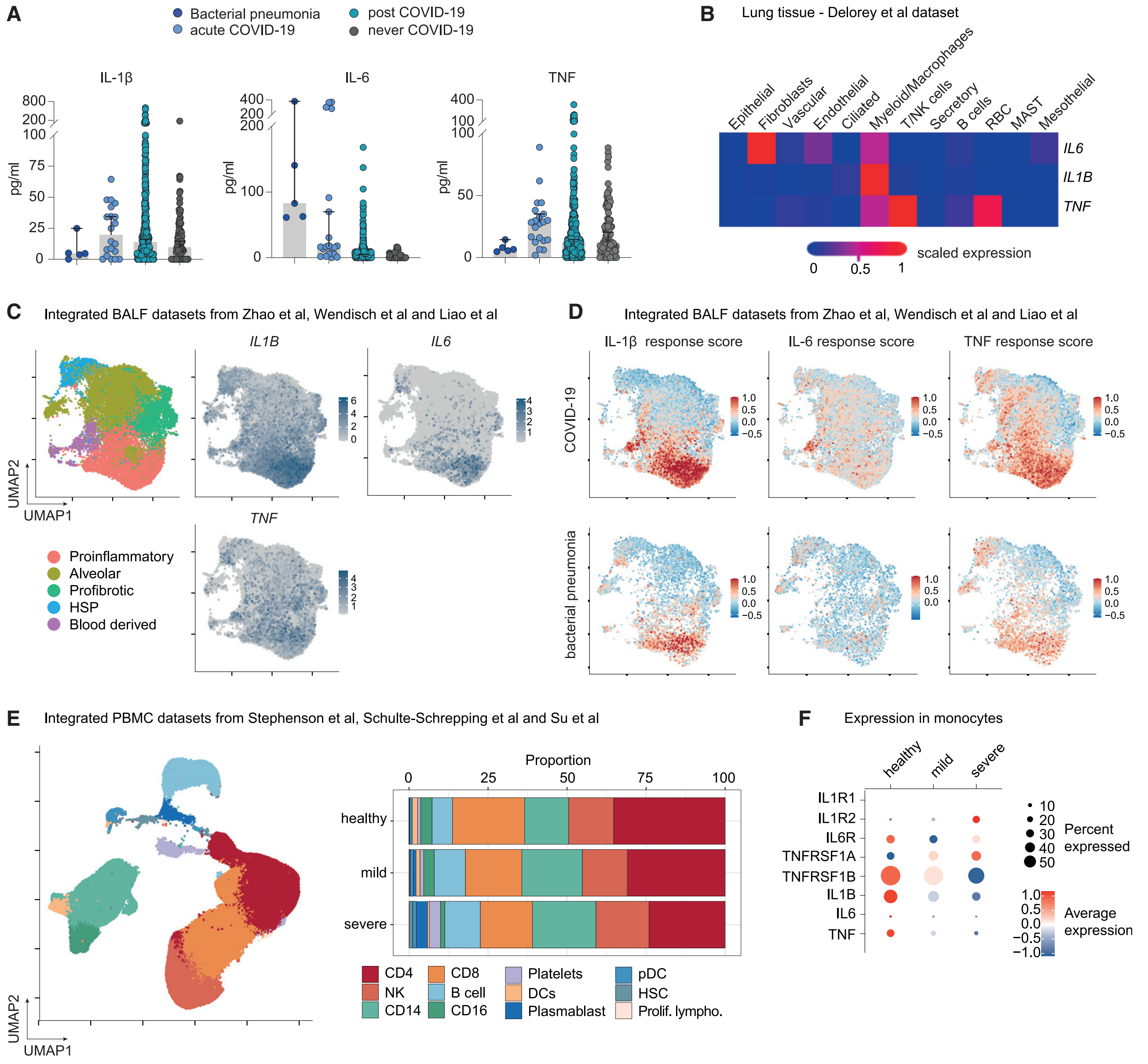

Figure 5. PASC-related cytokine triad in acute COVID-19 and profiling of *IL1B*, *IL6*, and *TNF* signatures in different tissues from hospitalized COVID-19 patients or individuals with mild to moderate COVID-19 courses

(A) Median plasma levels of IL-1β, IL6, and TNF in acute COVID-19 (n = 20) and in post-acute disease phases (n = 471) as compared with patients with bacterial pneumonia (n = 5) or individuals without prior COVID-19 (n = 96). Samples from patients with bacterial pneumonia and acute COVID-19 derived from the HACO trial; follow-up blood samples derived from DigiHero and the HACO trial. Bars indicate 95% confidence interval.

(B) Profiling of *IL1B*, *IL6*, and *TNF* transcripts in lung autopsy tissue from deceased COVID-19 patients. Single-cell transcriptome dataset from Delorey et al.[39]

(C) Macrophage subsets from bronchoalveolar (BAL) fluid in active COVID-19. Integrated single-cell dataset from Zhao et al.,[40] Wendisch et al.,[41] and Liao et al.[42] encompassing 19,089 cells. Uniform Manifold Approximation and Projection (UMAP) plot showing expression of *IL1B*, *IL6*, and *TNF* in macrophage subpopulations.

(D) Analysis of gene set associated with response to cytokine triad in macrophage subsets from bronchoalveolar fluid in active COVID-19.

(E) Generation of an integrated peripheral blood mononuclear cell (PBMC) dataset encompassing 39 healthy individuals (140,472 cells), 50 COVID-19 patients with mild (167,160 cells) and 19 with severe (81,754 cells) courses with data derived from Stephenson et al.,[43] Schulte-Schrepping et al.,[44] and Su et al.[45]

(F) Expression of *IL1B*, *IL6*, *TNF*, and their receptors *IL1R1*, *IL1R2*, *IL6R*, *TNFRSF1A*, and *TNFRSF1B* in monocytes relative to the remaining cells in the integrated datasets from (E) shown as a dotplot.

gene sets encompassing the respective cytokine receptors (*IL1R1*, *IL1R2*, *IL6R*, *TNFRSF1A*, *TNFRSF1B*) and diverse response genes. As shown in Figure 5D, BALF-derived macrophages from patients with COVID-19 show especially high response scores for IL-1β and TNF in their pro-inflammatory subset. A similar, albeit substantially weaker pattern is observed in macrophages from patients with bacterial pneumonia (Figure 5D). The inclusion of the late-phase TNF response genes *CLU*, *TNIP3*, *SIGLEC10*, *ENPP2*, and *NKG7*[48] in the TNF response score further supported a chronic, self-sustaining activation of pro-inflammatory macrophage. For IL-6, BALF macrophages of COVID-19 patients also exhibit elevated but generally lower response scores that are more scattered across subsets and have highest values in blood-derived monocytes (Figure 5D). Interestingly, macrophages from the bacterial pneumonia patients have only marginal response scores for IL-6 (Figure 5D), albeit having higher plasma levels (Figure 5A). Since BALF-derived macrophages are more representative for tissue infiltrating or resident cells combating infection *in situ*, we next asked whether the observed response patterns are also detectable in the blood. For this purpose, we generated an integrated dataset of peripheral blood mononuclear cells from COVID-19 patients with mild or severe courses as well as from healthy individuals[43–45] and profiled them for IL-1β, IL-6, and TNF-related gene expression. The proportion of peripheral CD14-expressing monocytes was found to be elevated in COVID-19 patients while the non-classical CD16 monocytes decrease (Figure 5E); yet these peripherally circulating monocytes did not clearly recapitulate the IL-1β, IL-6, and TNF signatures found in local monocytes/macrophages from the BALF samples (Figure 5F).

DISCUSSION

A considerable fraction of patients do not fully recover from COVID-19 but experience lasting sequelae. There are a number of recent population and registration studies addressing the long-term outcomes of patients with COVID-19 both with and without prior hospital admission. These studies suggest that approximately 32% to 87% of patients infected with SARS-CoV-2 develop PASC.[8,11,12,49,50] In addition to the inherently restricted follow-up of these studies, their major limitation is the lack of bioimmunological data acquired in subjects with PASC. The wealth of studies dissecting the biology of the acute COVID-19 infection dramatically contrasts with the paucity of biological data currently available for patients with persistent symptoms. As a consequence, current concepts of the natural history of PASC and its pathophysiological drivers remain hypothetical. Yet, these data are urgently needed to develop rational therapeutic strategies.

In the work presented here, we aimed to address these questions by analyzing the first 318 participants from the DigiHero cohort that follows an ambidirectional digital epidemiology approach with a survey focusing on the COVID-19 and vaccination history and a prospective part including an invitation to donate blood for (auto-)antibody and cytokine profiling. To confirm these results, we recruited a second validation cohort comprising another 333 DigiHero participants.

We found that 60% to 70% of previously infected participants experienced prolonged symptoms independent of age and were therefore categorized as affected by PASC. Approximately 27% to 40% of the initially infected subjects even showed ongoing symptoms at the time of blood sampling, which was on average 8 to 10 months after positive SARS-CoV-2 testing, including five individuals with persistent symptoms 16 to 24 months post-COVID-19, which is substantially longer than the durations reported so far by others.[8,11,49] Notably, after SARS-CoV or Middle East respiratory syndrome (MERS) infections, long-lasting post-acute sequelae including fatigue, pain, and psychiatric morbidities are also common for up to 36 months and reported as chronic post-SARS/MERS syndrome.[51–53] Similar to SARS-CoV and MERS and in line with other studies on SARS-CoV-2,[8,10] the most consistently reported PASC symptoms were fatigue, dyspnea, loss of taste and smell, and neurophysiological manifestations including sleep disturbances and lack of concentration. Our study confirms the higher proportion of PASC in women versus men.[8,49,50,54,55] This is not biased by the higher DigiHero response rate of women, since the percentages of PASC reporting individuals referred to all women or all men with prior COVID-19 enrolled in the DigiHero trial. In contrast, male sex has been clearly established as risk factor for severe COVID-19.[56,57] These observations are often discussed in the context of sex-specific immune responses and autoimmunity, e.g., females mounting stronger anti-viral responses but more often develop autoimmune disorders.[58] Our data do not provide an explanation for why women are more frequently affected by PASC, but it suggests that the pathobiological mechanisms underlying PASC in non-hospitalized patients may be similar in men and women.

One hypothetical mechanistic explanation for PASC could be inefficient clearing of SARS-CoV-2 resulting in cryptic viral reservoirs especially outside the lung. In this notion, vaccination-induced boosting of the SARS-CoV-2-directed immune response toward increased neutralizing breadth and potency could contribute to eradication of latent virus or its immunogenic remnants and thereby to the resolution of PASC. Indeed, while SARS-CoV-2 RNA shedding peaks around 10 to 14 days post-infection, persistence in serum and stool samples up to 126 days post-infection has been reported, similar to SARS-CoV and MERS.[59,60] In addition, viral RNA and protein were detected in the lower gastrointestinal tract in about 30% of tested individuals with negative nasopharyngeal-swab PCR up to 6 months post-infection.[18] Our digital cohort allowed us to assess the relationship between post-infection vaccination and resolution of PASC. These data show that the post-infection vaccination rate is equally high in participants in whom PASC eventually resolves as in those with ongoing PASC. Moreover, the percentage of participants with ongoing PASC was comparable in vaccinated and non-vaccinated subgroups. These data do not *prima facie* support the hypothesis of a cryptic SARS-CoV-2 reservoir underlying PASC that may be eradicated by a vaccination-boosted immune response. However, there are reports showing a reduced risk of developing PASC in individuals who received two vaccine doses before SARS-CoV-2 infection or one dose within the first weeks after infection.[12] Since participants in our cohort received vaccination not earlier than

3 months after a negative PCR test, one might speculate that there might be a short time window post-infection that can be exploited to refine an ongoing SARS-CoV-2-directed immune response and prevent chronification of immune dysregulation.

A striking feature of COVID-19 are systemic manifestations of autoimmunity mirrored by elevated levels of different autoantibody classes,[22–28,33] a feature that is shared by many viral infections including SARS-CoV and MERS.[61,62] While autoantibody positivity correlates with COVID-19 severity,[22–24,32,33] the potential pathophysiological relevance for PASC is unclear. A recently published study correlated PASC with autoantibodies targeting IFN-α2 and ANAs in a cohort consisting of predominantly hospitalized individuals.[63] In line with the two-hit model of COVID-19 immune pathogenesis that provides an explanation for acute severity of COVID-19,[33] this finding argues for persistent neutralization of IFN responses resulting in insufficient viral clearance, ongoing dysregulation of IFN-dependent immunity, and tissue damage, which is mirrored by ANA positivity. In our cohort, which did not comprise hospitalized patients and was collected substantially later, there was no detectable correlation of autoantibodies including ANAs with PASC as well as no evidence for newly emerging autoimmune conditions. The clinical relevance of such SARS-CoV-2-induced autoantibodies therefore still remains debatable and might depend on acute severity.

In contrast, our cytokine profiling revealed a significant association of a well-known triad of cytokines—IL-1β, IL-6, and TNF—with PASC. Due to their functional role in pain perception, anxiety, depression, and inflammatory symptoms[64–73] they are well compatible with the spectrum of symptoms in PASC. While the plasma levels of all three cytokines are highly elevated in acute COVID-19,[37,74,75] IL-6 in particular represents a key inflammatory driver of SARS-CoV-2-dependent immune pathogenesis as highlighted by its suitability as marker for severity and survival of COVID-19[76,77] and the benefit of IL-6-directed treatment in hospitalized patients when combined with corticosteroids as found by the REMAP-CAP[78] and RECOVERY[79] trials. It should be noted that IL-6 plasma levels in COVID-19 are lower as compared with other conditions like sepsis, acute respiratory distress syndrome (ARDS), or chimeric antigen receptor T cell-associated cytokine release syndrome (CAR-T CRS).[38] However, this does not necessarily reflect magnitude and duration of IL-6-mediated signaling, which is dependent on the complex interplay of membrane-bound (*cis*-signaling, classically anti-inflammatory) and soluble IL-6 receptors (*trans*-signaling, pro-inflammatory) as well as soluble inhibitors (sgp130).[38,80] The relevance of IL-6 *trans*-signaling for acute COVID-19 has recently been reported.[75] The important role for IL-6 in PASC development and persistence we postulate here is also substantiated by recent data from Phetsouphanh and colleagues[81] who monitored 31 PASC patients up to 8 months and identified a set of interferons and IL-6 as PASC classifiers with around 80% accuracy. In addition, higher IL-6 levels during acute COVID-19 correlate with PASC-associated anti-IFN-α2 autoantibodies[63] and radiological sequelae 3 months post-infection.[82]

Elevation of IL-1β, IL-6, and TNF are compatible with two of the currently most discussed hypotheses on the immune pathogenesis of PASC, which are ongoing immune responses against persisting virus or viral antigens and/or chronic reprogramming of immune cells. The persisting immune response hypothesis has been fueled by the finding of immunogenic viral particles in multiple tissues, including but not limited to the respiratory tract, kidneys, brain, and gastrointestinal and cardiac systems months after infection[12] and elevated levels of highly activated $CD38^+HLA\text{-}DR^+$ myeloid cells and $CD14^+CD16^+$ monocytes, which are also reported to contain persisting S1 proteins in PASC patients,[83] as well as plasmacytoid dendritic cells (pDCs) and several interferons in the blood of PASC patients.[81] In addition, the expansion of SARS-CoV-2-specific $CD8^+$ and $CD4^+$ T cell clonotypes during PASC has been reported.[63] In this notion, IL-1β, IL-6, and TNF can be readily interpreted as mediators of an ongoing SARS-CoV-2-directed immune response.

Nevertheless, given the plethora of deregulated cytokines in severe and mild cases of acute COVID-19, it appears interesting that only three cytokines clearly correlate with PASC. While it is plausible that other cytokines also contribute to the reported PASC symptoms on a less systemic level due to tissue restrictions of cryptic viral reservoirs, our data substantiate the concept of a persistent reprogramming of distinct pro-inflammatory immune cells. This concept suggests that the phase of uncontrolled self-fueled hyperinflammation in acute COVID-19 transitions into a state of persisting immune cell perturbations that drive PASC. In line with this model, we observe IL-1β, IL-6, and TNF elevations in post-acute disease phases that selectively persist in long-term PASC. Similar observations during early recovery have been reported in another cross-sectional study for IL-6 and TNF.[84] Analysis of macrophages from the BALF in patients with severe COVID-19 showed that a specific pro-inflammatory macrophage subset produced high levels of these cytokines in acute disease.[40,44] Mining of integrated single-cell sequencing for these cytokine response pathways revealed that such macrophages may not only be primed in the lung to produce the cytokine triad, but may also respond to it. Notably, these signatures were less homogeneous in the periphery. The nature of response genes involved suggested chronic cytokine exposure. Based on these data, one may speculate that acute pro-inflammatory reprogramming of long-lived lung macrophages or their precursors may result in a vicious circle of IL-1β, IL-6, and TNF production that self-maintains this cellular compartment. This hypothesis may be supported by recent evidence showing reprogramming of the monocyte/macrophage compartment in COVID-19 that results in pathological inflammasome engagement in these cells.[85] In addition, a long-term inflammatory memory is imprinted on the monocyte/macrophage compartment driving aberrant effector functions and eicosanoid metabolism.[86] It is noteworthy that these imprints are related to increased levels of IL-1β, IL-6, and TNF secretion upon stimulation and are so far mainly observed in non-hospitalized patients with mild to moderate courses. This is in line with the observation that blood-derived monocytes from mild but not from severe patients secrete IL-1β and TNF.[44] Yet, to definitively prove the causal relationship between the cytokine triad and self-sustained, chronic activation of reprogrammed macrophage in patients with PASC, matched tissue and blood analysis at the PASC stage would be instrumental.

Finally, the PASC-associated long-term elevation of IL-1β, IL-6, and TNF plasma levels opens up therapeutic options.

The effectiveness of IL-6 targeting in combination with corticosteroids was already shown for severe cases of acute COVID-19,[78,79] and TNF blockade might be beneficial in acute COVID-19 as suggested by small observational case studies,[87] in addition to its long history as therapeutic for a variety of rheumatic diseases characterized by chronic TNF elevations. In contrast, blocking of IL-1 signaling with anakinra had no substantial effect on reducing mortality of critically ill COVID-19 patients.[88,89] However, there have been reports that long-term metformin users have a strikingly reduced risk of fatal courses,[90,91] with one study showing that this is especially true for women with obesity or type 2 diabetes.[92] It was recently shown that metformin blocks NLRP3 inflammasome activation including inhibition of IL-1β and IL-6 in alveolar macrophages attenuating SARS-CoV-2-induced ARDS in a mouse model.[93] Based on these data, one might speculate that inhibition of IL-1β production might outperform receptor blockade in the context of hyperinflammatory ARDS.

Taken together, we report chronic elevation of IL-1β, IL-6, and TNF plasma levels in PASC. The combination of digital epidemiology and selective biobanking put us in a position to rapidly explore biomarkers of PASC in a well-characterized cohort of patients with a considerable follow-up of 8 to 10 months. It is noteworthy, that, owing to the digital recruitment approach, the recruitment time was only 2 weeks from invitation. Since participants answered the questionnaires online, identification of eligible participants for the COVID-19 module was possible in real time. Digitalization may therefore considerably accelerate epidemiological health research, which is of use not only for pandemic research questions.

Limitations of the study

A limitation of this study is the "open invitation" study design and the self-reporting of PASC symptoms that might bias the analysis, since participants with ongoing symptoms and higher subjective symptom load may be selected.[94] Nevertheless, other studies with different designs reported equivalent PASC proportions ranging from 55% to 87% of participating individuals.[82,95–97] Noteworthy in this regard is especially the study from Huang and colleagues[97] that assessed post-acute sequelae in 86% of all laboratory-confirmed COVID-19 patients discharged from Jin Yin-tan Hospital between January and May 2020, finding PASC symptoms in 76%. In addition, it remains to be determined which cells in which tissue are the main source for the detected cytokines and whether their secretion is triggered by viral remnants or mediated by a COVID-19-induced reprogramming.

STAR★METHODS

Detailed methods are provided in the online version of this paper and include the following:

- KEY RESOURCES TABLE
- RESOURCE AVAILABILITY
  - ○ Lead contact
  - ○ Materials availability
  - ○ Data and code availability
- EXPERIMENTAL MODEL AND SUBJECT DETAILS
  - ○ COVID-19 module within the DigiHero cohort study (population-based cohort study for digital health research in Germany)
  - ○ Biological samples and data from the biobank of the halle COVID cohort (HACO)
- METHOD DETAILS
  - ○ SARS-CoV-2 antibody profiling
  - ○ Cytokine and autoantibody profiling
  - ○ Single-cell transcriptome analyzes
- QUANTIFICATION AND STATISTICAL ANALYSIS

SUPPLEMENTAL INFORMATION

Supplemental information can be found online at https://doi.org/10.1016/j.xcrm.2022.100663.

ACKNOWLEDGMENTS

The DigiHero study is conducted by a consortium of the Medical Faculty of the Martin-Luther-University Halle-Wittenberg, including the following PIs: Mascha Binder, Thomas Frese, Michael Gekle, Matthias Girndt, Jessica Höll, Patrick Michl, Rafael Mikolajczyk, Matthias Richter, and Daniel Sedding. We thank Aline Patzschke, Christoph Wosiek, and Bianca Gebhardt for excellent technical assistance. We sincerely thank healthy donors, patients, and their household members for participating in this study. Flow cytometry was performed at the UKH FACS sorting core facility. This project was partially funded by the CRC 841 of the German Research Foundation (to M.B.), as well as by the Medical Faculty of the Martin-Luther-University Halle-Wittenberg. We acknowledge the financial support of the Open Access Publication Fund of the Martin-Luther-University Halle-Wittenberg.

AUTHOR CONTRIBUTIONS

M.B., R.M., M.G., C.S., L.P., S.H., C.G., B.K., M.G., T.F., D.S., and J.H. designed the COVID-19 module of the DigiHero cohort study. D.S. and J.D. provided the HACO patient cohort. C.S. and L.P. conducted experiments. M.B., C.S., L.P., E.W., and L.B. analyzed and interpreted the data. M.B., C.S., E.W., and L.P. drafted the manuscript. All authors critically revised and approved the manuscript.

DECLARATION OF INTERESTS

The authors declare no competing interests.

Received: January 11, 2022
Revised: March 28, 2022
Accepted: May 25, 2022
Published: June 21, 2022

REFERENCES

1. Salian, V.S., Wright, J.A., Vedell, P.T., Nair, S., Li, C., Kandimalla, M., Tang, X., Carmona Porquera, E.M., Kalari, K.R., and Kandimalla, K.K. (2021). COVID-19 transmission, current treatment, and future therapeutic strategies. Mol. Pharm. *18*, 754–771. https://doi.org/10.1021/acs.molpharmaceut.0c00608.
2. Kumar, A., Singh, R., Kaur, J., Pandey, S., Sharma, V., Thakur, L., Sati, S., Mani, S., Asthana, S., Sharma, T.K., et al. (2021). Wuhan to world: the COVID-19 pandemic. Front. Cell. Infect. Microbiol. *11*, 596201. https://doi.org/10.3389/fcimb.2021.596201.
3. Ramos-Casals, M., Brito-Zerón, P., and Mariette, X. (2021). Systemic and organ-specific immune-related manifestations of COVID-19. Nat. Rev. Rheumatol. *17*, 315–332. https://doi.org/10.1038/s41584-021-00608-z.

CellPress
OPEN ACCESS

4. Osuchowski, M.F., Winkler, M.S., Skirecki, T., Cajander, S., Shankar-Hari, M., Lachmann, G., Monneret, G., Venet, F., Bauer, M., Brunkhorst, F.M., et al. (2021). The COVID-19 puzzle: deciphering pathophysiology and phenotypes of a new disease entity. Lancet Respir. Med. *9*, 622–642. https://doi.org/10.1016/S2213-2600(21)00218-6.
5. Bartleson, J.M., Radenkovic, D., Covarrubias, A.J., Furman, D., Winer, D.A., and Verdin, E. (2021). SARS-CoV-2, COVID-19 and the aging immune system. Nat. Aging *1*, 769–782. https://doi.org/10.1038/s43587-021-00114-7.
6. Proal, A.D., and VanElzakker, M.B. (2021). Long COVID or post-acute sequelae of COVID-19 (PASC): an overview of biological factors that may contribute to persistent symptoms. Front. Microbiol. *12*, 698169. https://doi.org/10.3389/fmicb.2021.698169.
7. Groff, D., Sun, A., Ssentongo, A.E., Ba, D.M., Parsons, N., Poudel, G.R., Lekoubou, A., Oh, J.S., Ericson, J.E., Ssentongo, P., and Chinchilli, V.M. (2021). Short-term and long-term rates of postacute sequelae of SARS-CoV-2 infection: a systematic review. JAMA Netw. Open *4*, e2128568. https://doi.org/10.1001/jamanetworkopen.2021.28568.
8. Nalbandian, A., Sehgal, K., Gupta, A., Madhavan, M.V., McGroder, C., Stevens, J.S., Cook, J.R., Nordvig, A.S., Shalev, D., Sehrawat, T.S., et al. (2021). Post-acute COVID-19 syndrome. Nat. Med. *27*, 601–615. https://doi.org/10.1038/s41591-021-01283-z.
9. Bell, M.L., Catalfamo, C.J., Farland, L.V., Ernst, K.C., Jacobs, E.T., Klimentidis, Y.C., Jehn, M., and Pogreba-Brown, K. (2021). Post-acute sequelae of COVID-19 in a non-hospitalized cohort: results from the Arizona CoVHORT. PLoS One *16*, e0254347. https://doi.org/10.1371/journal.pone.0254347.
10. Sudre, C.H., Murray, B., Varsavsky, T., Graham, M.S., Penfold, R.S., Bowyer, R.C., Pujol, J.C., Klaser, K., Antonelli, M., Canas, L.S., et al. (2021). Attributes and predictors of long COVID. Nat. Med. *27*, 626–631. https://doi.org/10.1038/s41591-021-01292-y.
11. Mehandru, S., and Merad, M. (2022). Pathological sequelae of long-haul COVID. Nat. Immunol. *23*, 194–202. https://doi.org/10.1038/s41590-021-01104-y.
12. Merad, M., Blish, C.A., Sallusto, F., and Iwasaki, A. (2022). The immunology and immunopathology of COVID-19. Science *375*, 1122–1127. https://doi.org/10.1126/science.abm8108.
13. Guler, S.A., Ebner, L., Aubry-Beigelman, C., Bridevaux, P.O., Brutsche, M., Clarenbach, C., Garzoni, C., Geiser, T.K., Lenoir, A., Mancinetti, M., et al. (2021). Pulmonary function and radiological features 4 months after COVID-19: first results from the national prospective observational Swiss COVID-19 lung study. Eur. Respir. J. *57*, 2003690. https://doi.org/10.1183/13993003.03690-2020.
14. Rai, D.K., Sharma, P., and Kumar, R. (2021). Post covid 19 pulmonary fibrosis. Is it real threat? Indian J. Tuberc. *68*, 330–333. https://doi.org/10.1016/j.ijtb.2020.11.003.
15. Liotti, F.M., Menchinelli, G., Marchetti, S., Posteraro, B., Landi, F., Sanguinetti, M., and Cattani, P. (2021). Assessment of SARS-CoV-2 RNA test results among patients who recovered from COVID-19 with prior negative results. JAMA Intern. Med. *181*, 702. https://doi.org/10.1001/jamainternmed.2020.7570.
16. Sun, J., Xiao, J., Sun, R., Tang, X., Liang, C., Lin, H., Zeng, L., Hu, J., Yuan, R., Zhou, P., et al. (2020). Prolonged persistence of SARS-CoV-2 RNA in body fluids. Emerg. Infect. Dis. *26*, 1834–1838. https://doi.org/10.3201/eid2608.201097.
17. Vibholm, L.K., Nielsen, S.S.F., Pahus, M.H., Frattari, G.S., Olesen, R., Andersen, R., Monrad, I., Andersen, A.H.F., Thomsen, M.M., Konrad, C.V., et al. (2021). SARS-CoV-2 persistence is associated with antigen-specific CD8 T-cell responses. eBioMedicine *64*, 103230. https://doi.org/10.1016/j.ebiom.2021.103230.
18. Gaebler, C., Wang, Z., Lorenzi, J.C.C., Muecksch, F., Finkin, S., Tokuyama, M., Cho, A., Jankovic, M., Schaefer-Babajew, D., Oliveira, T.Y., et al. (2021). Evolution of antibody immunity to SARS-CoV-2. Nature *591*, 639–644. https://doi.org/10.1038/s41586-021-03207-w.
19. Randall, R.E., and Griffin, D.E. (2017). Within host RNA virus persistence: mechanisms and consequences. Curr. Opin. Virol. *23*, 35–42. https://doi.org/10.1016/j.coviro.2017.03.001.
20. Massey, D., Berrent, D., Akrami, A., Assaf, G., Davis, H., Harris, K., McCorkell, L., Ring, A.M., Schulz, W.L., Wei, H., et al. (2021). Change in symptoms and immune response in people with post-acute sequelae of SARS-Cov-2 infection (PASC) after SARS-Cov-2 vaccination. Preprint at medRxiv. https://doi.org/10.1101/2021.07.21.21260391.
21. Woodruff, M.C., Ramonell, R.P., Nguyen, D.C., Cashman, K.S., Saini, A.S., Haddad, N.S., Ley, A.M., Kyu, S., Howell, J.C., Ozturk, T., et al. (2020). Extrafollicular B cell responses correlate with neutralizing antibodies and morbidity in COVID-19. Nat. Immunol. *21*, 1506–1516. https://doi.org/10.1038/s41590-020-00814-z.
22. Schultheiß, C., Paschold, L., Willscher, E., Simnica, D., Wöstemeier, A., Muscate, F., Wass, M., Eisenmann, S., Dutzmann, J., Keyßer, G., et al. (2021). Maturation trajectories and transcriptional landscape of plasmablasts and autoreactive B cells in COVID-19. iScience *24*, 103325. https://doi.org/10.1016/j.isci.2021.103325.
23. Bastard, P., Gervais, A., Le Voyer, T., Rosain, J., Philippot, Q., Manry, J., Michailidis, E., Hoffmann, H.H., Eto, S., Garcia-Prat, M., et al. (2021). Autoantibodies neutralizing type I IFNs are present in ~4% of uninfected individuals over 70 years old and account for ~20% of COVID-19 deaths. Sci. Immunol. *6*, eabl4340. https://doi.org/10.1126/sciimmunol.abl4340.
24. Wang, E.Y., Mao, T., Klein, J., Dai, Y., Huck, J.D., Jaycox, J.R., Liu, F., Zhou, T., Israelow, B., Wong, P., et al. (2021). Diverse functional autoantibodies in patients with COVID-19. Nature *595*, 283–288. https://doi.org/10.1038/s41586-021-03631-y.
25. Borghi, M.O., Beltagy, A., Garrafa, E., Curreli, D., Cecchini, G., Bodio, C., Grossi, C., Blengino, S., Tincani, A., Franceschini, F., et al. (2020). Anti-phospholipid antibodies in COVID-19 are different from those detectable in the anti-phospholipid syndrome. Front. Immunol. *11*, 584241. https://doi.org/10.3389/fimmu.2020.584241.
26. El Hasbani, G., Taher, A.T., Jawad, A., and Uthman, I. (2020). COVID-19, antiphospholipid antibodies, and Catastrophic antiphospholipid syndrome: a possible association? Clin. Med. Insights Arthritis Musculoskelet. Disord. *13*, 117954412097866. https://doi.org/10.1177/1179544120978667.
27. Vlachoyiannopoulos, P.G., Magira, E., Alexopoulos, H., Jahaj, E., Theophilopoulou, K., Kotanidou, A., and Tzioufas, A.G. (2020). Autoantibodies related to systemic autoimmune rheumatic diseases in severely ill patients with COVID-19. Ann. Rheum. Dis. *79*, 1661–1663. https://doi.org/10.1136/annrheumdis-2020-218009.
28. Zuo, Y., Estes, S.K., Ali, R.A., Gandhi, A.A., Yalavarthi, S., Shi, H., Sule, G., Gockman, K., Madison, J.A., Zuo, M., et al. (2020). Prothrombotic autoantibodies in serum from patients hospitalized with COVID-19. Sci. Transl. Med. *12*, eabd3876. https://doi.org/10.1126/scitranslmed.abd3876.
29. Porritt, R.A., Binek, A., Paschold, L., Rivas, M.N., McArdle, A., Yonker, L.M., Alter, G., Chandnani, H.K., Lopez, M., Fasano, A., et al. (2021). The autoimmune signature of hyperinflammatory multisystem inflammatory syndrome in children. J. Clin. Invest. *131*, e151520. https://doi.org/10.1172/JCI151520.
30. Liu, Y., Sawalha, A.H., and Lu, Q. (2021). COVID-19 and autoimmune diseases. Curr. Opin. Rheumatol. *33*, 155–162. https://doi.org/10.1097/BOR.0000000000000776.
31. Ahmed, S., Zimba, O., and Gasparyan, A.Y. (2021). COVID-19 and the clinical course of rheumatic manifestations. Clin. Rheumatol. *40*, 2611–2619. https://doi.org/10.1007/s10067-021-05691-x.
32. Knight, J.S., Caricchio, R., Casanova, J.L., Combes, A.J., Diamond, B., Fox, S.E., Hanauer, D.A., James, J.A., Kanthi, Y., Ladd, V., et al. (2021). The intersection of COVID-19 and autoimmunity. J. Clin. Invest. *131*, e154886. https://doi.org/10.1172/jci154886.
33. Zhang, Q., Bastard, P., Karbuz, A., Gervais, A., Tayoun, A.A., Aiuti, A., Belot, A., Bolze, A., Gaudet, A., Bondarenko, A., et al. (2022). Human genetic and immunological determinants of critical COVID-19 pneumonia. Nature *603*, 587–598. https://doi.org/10.1038/s41586-022-04447-0.

34. Andreano, E., Paciello, I., Piccini, G., Manganaro, N., Pileri, P., Hyseni, I., Leonardi, M., Pantano, E., Abbiento, V., Benincasa, L., et al. (2021). Hybrid immunity improves B cells and antibodies against SARS-CoV-2 variants. Nature *600*, 530–535. https://doi.org/10.1038/s41586-021-04117-7.

35. Wilson, C. (2021). Vaccines may help clear up long-term covid-19 symptoms. New Sci. *249*, 9. https://doi.org/10.1016/S0262-4079(21)00396-1.

36. Paschold, L., Klee, B., Gottschick, C., Willscher, E., Diexer, S., Schultheiß, C., Simnica, D., Sedding, D., Girndt, M., Gekle, M., et al. (2022). Rapid hypermutation B cell trajectory recruits previously primed B cells upon third SARS-Cov-2 mRNA vaccination. Front. Immunol. *13*, 876306. https://doi.org/10.3389/fimmu.2022.876306.

37. Schultheiß, C., Paschold, L., Simnica, D., Mohme, M., Willscher, E., von Wenserski, L., Scholz, R., Wieters, I., Dahlke, C., Tolosa, E., et al. (2020). Next-generation sequencing of T and B cell receptor repertoires from COVID-19 patients showed signatures associated with severity of disease. Immunity *53*, 442–455.e4. https://doi.org/10.1016/j.immuni.2020.06.024.

38. Chen, L.Y.C., Biggs, C.M., Jamal, S., Stukas, S., Wellington, C.L., and Sekhon, M.S. (2021). Soluble interleukin-6 receptor in the COVID-19 cytokine storm syndrome. Cell Rep. Med. *2*, 100269. https://doi.org/10.1016/j.xcrm.2021.100269.

39. Delorey, T.M., Ziegler, C.G.K., Heimberg, G., Normand, R., Yang, Y., Segerstolpe, Å., Abbondanza, D., Fleming, S.J., Subramanian, A., Montoro, D.T., et al. (2021). COVID-19 tissue atlases reveal SARS-CoV-2 pathology and cellular targets. Nature *595*, 107–113. https://doi.org/10.1038/s41586-021-03570-8.

40. Zhao, Y., Kilian, C., Turner, J.E., Bosurgi, L., Roedl, K., Bartsch, P., Gnirck, A.C., Cortesi, F., Schultheiß, C., Hellmig, M., et al. (2021). Clonal expansion and activation of tissue-resident memory-like Th17 cells expressing GM-CSF in the lungs of severe COVID-19 patients. Sci. Immunol. *6*, eabf6692. https://doi.org/10.1126/sciimmunol.abf6692.

41. Wendisch, D., Dietrich, O., Mari, T., von Stillfried, S., Ibarra, I.L., Mittermaier, M., Mache, C., Chua, R.L., Knoll, R., Timm, S., et al. (2021). SARS-CoV-2 infection triggers profibrotic macrophage responses and lung fibrosis. Cell *184*, 6243–6261.e27. https://doi.org/10.1016/j.cell.2021.11.033.

42. Liao, M., Liu, Y., Yuan, J., Wen, Y., Xu, G., Zhao, J., Cheng, L., Li, J., Wang, X., Wang, F., et al. (2020). Single-cell landscape of bronchoalveolar immune cells in patients with COVID-19. Nat. Med. *26*, 842–844. https://doi.org/10.1038/s41591-020-0901-9.

43. Stephenson, E., Reynolds, G., Botting, R.A., Calero-Nieto, F.J., Morgan, M.D., Tuong, Z.K., Bach, K., Sungnak, W., Worlock, K.B., Yoshida, M., et al. (2021). Single-cell multi-omics analysis of the immune response in COVID-19. Nat. Med. *27*, 904–916. https://doi.org/10.1038/s41591-021-01329-2.

44. Schulte-Schrepping, J., Reusch, N., Paclik, D., Baßler, K., Schlickeiser, S., Zhang, B., Krämer, B., Krammer, T., Brumhard, S., Bonaguro, L., et al. (2020). Severe COVID-19 is marked by a dysregulated myeloid cell compartment. Cell *182*, 1419–1440.e23. https://doi.org/10.1016/j.cell.2020.08.001.

45. Su, Y., Chen, D., Yuan, D., Lausted, C., Choi, J., Dai, C.L., Voillet, V., Duvvuri, V.R., Scherler, K., Troisch, P., et al. (2020). Multi-Omics resolves a sharp disease-state shift between mild and moderate COVID-19. Cell *183*, 1479–1495.e20. https://doi.org/10.1016/j.cell.2020.10.037.

46. Arango Duque, G., and Descoteaux, A. (2014). Macrophage cytokines: involvement in immunity and infectious diseases. Front. Immunol. *5*, 491. https://doi.org/10.3389/fimmu.2014.00491.

47. Chua, R.L., Lukassen, S., Trump, S., Hennig, B.P., Wendisch, D., Pott, F., Debnath, O., Thürmann, L., Kurth, F., Völker, M.T., et al. (2020). COVID-19 severity correlates with airway epithelium-immune cell interactions identified by single-cell analysis. Nat. Biotechnol. *38*, 970–979. https://doi.org/10.1038/s41587-020-0602-4.

48. Huynh, L., Kusnadi, A., Park, S.H., Murata, K., Park-Min, K.H., and Ivashkiv, L.B. (2016). Opposing regulation of the late phase TNF response by mTORC1-IL-10 signaling and hypoxia in human macrophages. Sci. Rep. *6*, 31959. https://doi.org/10.1038/srep31959.

49. Seeßle, J., Waterboer, T., Hippchen, T., Simon, J., Kirchner, M., Lim, A., Müller, B., and Merle, U. (2021). Persistent symptoms in adult patients 1 Year after coronavirus disease 2019 (COVID-19): a prospective cohort study. Clin. Infect. Dis. *74*, 1191–1198. https://doi.org/10.1093/cid/ciab611.

50. Evans, R.A., McAuley, H., Harrison, E.M., Shikotra, A., Singapuri, A., Sereno, M., Elneima, O., Docherty, A.B., Lone, N.I., Leavy, O.C., et al. (2021). Physical, cognitive, and mental health impacts of COVID-19 after hospitalisation (PHOSP-COVID): a UK multicentre, prospective cohort study. Lancet Respir. Med. *9*, 1275–1287. https://doi.org/10.1016/S2213-2600(21)00383-0.

51. Moldofsky, H., and Patcai, J. (2011). Chronic widespread musculoskeletal pain, fatigue, depression and disordered sleep in chronic post-SARS syndrome; a case-controlled study. BMC Neurol. *11*, 37. https://doi.org/10.1186/1471-2377-11-37.

52. Mak, I.W., Chu, C.M., Pan, P.C., Yiu, M.G., and Chan, V.L. (2009). Long-term psychiatric morbidities among SARS survivors. Gen. Hosp. Psychiatry *31*, 318–326. https://doi.org/10.1016/j.genhosppsych.2009.03.001.

53. Ahmed, H., Patel, K., Greenwood, D.C., Halpin, S., Lewthwaite, P., Salawu, A., Eyre, L., Eyre, L., O'Connor, R., O'Connor, R., et al. (2020). Long-term clinical outcomes in survivors of severe acute respiratory syndrome and Middle East respiratory syndrome coronavirus outbreaks after hospitalisation or ICU admission: a systematic review and meta-analysis. J. Rehabil. Med. *52*, jrm00063. https://doi.org/10.2340/16501977-2694.

54. Ganesh, R., Grach, S.L., Ghosh, A.K., Bierle, D.M., Salonen, B.R., Collins, N.M., Joshi, A.Y., Boeder, N.D., Jr., Anstine, C.V., Mueller, M.R., et al. (2022). The female-predominant persistent immune dysregulation of the post-COVID syndrome. Mayo Clin. Proc. *97*, 454–464. https://doi.org/10.1016/j.mayocp.2021.11.033.

55. Bai, F., Tomasoni, D., Falcinella, C., Barbanotti, D., Castoldi, R., Mulè, G., Augello, M., Mondatore, D., Allegrini, M., Cona, A., et al. (2022). Female gender is associated with long COVID syndrome: a prospective cohort study. Clin. Microbiol. Infect. *28*, 611.e9–611.e16. https://doi.org/10.1016/j.cmi.2021.11.002.

56. Bechmann, N., Barthel, A., Schedl, A., Herzig, S., Varga, Z., Gebhard, C., Mayr, M., Hantel, C., Beuschlein, F., Wolfrum, C., et al. (2022). Sexual dimorphism in COVID-19: potential clinical and public health implications. Lancet Diabetes Endocrinol. *10*, 221–230. https://doi.org/10.1016/S2213-8587(21)00346-6.

57. Bunders, M.J., and Altfeld, M. (2020). Implications of sex differences in immunity for SARS-CoV-2 pathogenesis and design of therapeutic Interventions. Immunity *53*, 487–495. https://doi.org/10.1016/j.immuni.2020.08.003.

58. Klein, S.L., and Flanagan, K.L. (2016). Sex differences in immune responses. Nat. Rev. Immunol. *16*, 626–638. https://doi.org/10.1038/nri.2016.90.

59. Phillips, S.P., Wei, X., Kwong, J.C., Gubbay, J., Schwartz, K.L., Majury, A., and Groome, P.A. (2021). Duration of SARS-CoV-2 shedding: a population-based, Canadian study. PLoS One *16*, e0252217. https://doi.org/10.1371/journal.pone.0252217.

60. Cevik, M., Tate, M., Lloyd, O., Maraolo, A.E., Schafers, J., and Ho, A. (2021). SARS-CoV-2, SARS-CoV, and MERS-CoV viral load dynamics, duration of viral shedding, and infectiousness: a systematic review and meta-analysis. Lancet Microbe *2*, e13–e22. https://doi.org/10.1016/S2666-5247(20)30172-5.

61. Wucherpfennig, K.W. (2001). Mechanisms for the induction of autoimmunity by infectious agents. J. Clin. Invest. *108*, 1097–1104. https://doi.org/10.1172/jci200114235.

62. Chakravarty, E.F. (2008). Viral infection and reactivation in autoimmune disease. Arthritis Rheum. *58*, 2949–2957. https://doi.org/10.1002/art.23883.

CellPress
OPEN ACCESS

63. Su, Y., Yuan, D., Chen, D.G., Ng, R.H., Wang, K., Choi, J., Li, S., Hong, S., Zhang, R., Xie, J., et al. (2022). Multiple early factors anticipate post-acute COVID-19 sequelae. Cell *185*, 881–895.e20. https://doi.org/10.1016/j.cell.2022.01.014.

64. Simen, B.B., Duman, C.H., Simen, A.A., and Duman, R.S. (2006). TNFα signaling in depression and anxiety: behavioral consequences of individual receptor targeting. Biol. Psychiatry *59*, 775–785. https://doi.org/10.1016/j.biopsych.2005.10.013.

65. Berthold-Losleben, M., and Himmerich, H. (2008). The TNFalpha system: functional aspects in depression, narcolepsy and psychopharmacology. Curr. Neuropharmacol. *6*, 193–202. https://doi.org/10.2174/157015908785777238.

66. Leung, L., and Cahill, C.M. (2010). TNF-alpha and neuropathic pain–a review. J. Neuroinflammation *7*, 27. https://doi.org/10.1186/1742-2094-7-27.

67. Atzeni, F., Nucera, V., Masala, I.F., Sarzi-Puttini, P., and Bonitta, G. (2019). Il-6 Involvement in pain, fatigue and mood disorders in rheumatoid arthritis and the effects of Il-6 inhibitor sarilumab. Pharmacol. Res. *149*, 104402. https://doi.org/10.1016/j.phrs.2019.104402.

68. Zhou, Y.Q., Liu, Z., Liu, Z.H., Chen, S.P., Li, M., Shahveranov, A., Ye, D.W., and Tian, Y.K. (2016). Interleukin-6: an emerging regulator of pathological pain. J. Neuroinflammation *13*, 141. https://doi.org/10.1186/s12974-016-0607-6.

69. O'Donovan, A., Hughes, B.M., Slavich, G.M., Lynch, L., Cronin, M.T., O'Farrelly, C., and Malone, K.M. (2010). Clinical anxiety, cortisol and interleukin-6: evidence for specificity in emotion-biology relationships. Brain Behav. Immun. *24*, 1074–1077. https://doi.org/10.1016/j.bbi.2010.03.003.

70. Niraula, A., Witcher, K.G., Sheridan, J.F., and Godbout, J.P. (2019). Interleukin-6 induced by social stress promotes a unique transcriptional signature in the monocytes that facilitate anxiety. Biol. Psychiatry *85*, 679–689. https://doi.org/10.1016/j.biopsych.2018.09.030.

71. Goshen, I., Kreisel, T., Ben-Menachem-Zidon, O., Licht, T., Weidenfeld, J., Ben-Hur, T., and Yirmiya, R. (2008). Brain interleukin-1 mediates chronic stress-induced depression in mice via adrenocortical activation and hippocampal neurogenesis suppression. Mol. Psychiatry *13*, 717–728. https://doi.org/10.1038/sj.mp.4002055.

72. Ren, K., and Torres, R. (2009). Role of interleukin-1β during pain and inflammation. Brain Res. Rev. *60*, 57–64. https://doi.org/10.1016/j.brainresrev.2008.12.020.

73. Rossi, S., Sacchetti, L., Napolitano, F., De Chiara, V., Motta, C., Studer, V., Musella, A., Barbieri, F., Bari, M., Bernardi, G., et al. (2012). Interleukin-1 Causes anxiety by Interacting with the endocannabinoid system. J. Neurosci. *32*, 13896–13905. https://doi.org/10.1523/JNEUROSCI.1515-12.2012.

74. Takahashi, T., Ellingson, M.K., Wong, P., Israelow, B., Lucas, C., Klein, J., Silva, J., Mao, T., Oh, J.E., Tokuyama, M., et al. (2020). Sex differences in immune responses that underlie COVID-19 disease outcomes. Nature *588*, 315–320. https://doi.org/10.1038/s41586-020-2700-3.

75. Koutsakos, M., Rowntree, L.C., Hensen, L., Chua, B.Y., van de Sandt, C.E., Habel, J.R., Zhang, W., Jia, X., Kedzierski, L., Ashhurst, T.M., et al. (2021). Integrated immune dynamics define correlates of COVID-19 severity and antibody responses. Cell Rep. Med. *2*, 100208. https://doi.org/10.1016/j.xcrm.2021.100208.

76. Del Valle, D.M., Kim-Schulze, S., Huang, H.H., Beckmann, N.D., Nirenberg, S., Wang, B., Lavin, Y., Swartz, T.H., Madduri, D., Stock, A., et al. (2020). An inflammatory cytokine signature predicts COVID-19 severity and survival. Nat. Med. *26*, 1636–1643. https://doi.org/10.1038/s41591-020-1051-9.

77. Woo, M.S., Haag, F., Nierhaus, A., Jarczak, D., Roedl, K., Mayer, C., Brehm, T.T., van der Meirschen, M., Hennigs, A., Christopeit, M., et al. (2021). Multi-dimensional and longitudinal systems profiling reveals predictive pattern of severe COVID-19. iScience *24*, 102752. https://doi.org/10.1016/j.isci.2021.102752.

78. Investigators, R.-C., Gordon, A.C., Mouncey, P.R., Al-Beidh, F., Rowan, K.M., Nichol, A.D., Arabi, Y.M., Annane, D., Beane, A., van Bentum-Puijk, W., et al. (2021). Interleukin-6 receptor antagonists in critically ill patients with covid-19. N. Engl. J. Med. *384*, 1491–1502. https://doi.org/10.1056/NEJMoa2100433.

79. Ghosn, L., Chaimani, A., Evrenoglou, T., Davidson, M., Graña, C., Schmucker, C., Bollig, C., Henschke, N., Sguassero, Y., Nejstgaard, C.H., et al. (2021). Interleukin-6 blocking agents for treating COVID-19: a living systematic review. Cochrane Database Syst. Rev. *3*, CD013881. https://doi.org/10.1002/14651858.CD013881.

80. Rose-John, S. (2012). IL-6 trans-signaling via the soluble IL-6 receptor: importance for the pro-inflammatory activities of IL-6. Int. J. Biol. Sci. *8*, 1237–1247. https://doi.org/10.7150/ijbs.4989.

81. Phetsouphanh, C., Darley, D.R., Wilson, D.B., Howe, A., Munier, C.M.L., Patel, S.K., Juno, J.A., Burrell, L.M., Kent, S.J., Dore, G.J., et al. (2022). Immunological dysfunction persists for 8 months following initial mild-to-moderate SARS-CoV-2 infection. Nat. Immunol. *23*, 210–216. https://doi.org/10.1038/s41590-021-01113-x.

82. Toraldo, D.M., Satriano, F., Rollo, R., Verdastro, G., Imbriani, G., Rizzo, E., Argentiero, A., Falco, A., Ambrosino, P., Miani, A., and Piscitelli, P. (2022). COVID-19 IgG/IgM patterns, early IL-6 elevation and long-term radiological sequelae in 75 patients hospitalized due to interstitial pneumonia followed up from 3 to 12 months. PLoS One *17*, e0262911. https://doi.org/10.1371/journal.pone.0262911.

83. Patterson, B.K., Francisco, E.B., Yogendra, R., Long, E., Pise, A., Rodrigues, H., Hall, E., Herrera, M., Parikh, P., Guevara-Coto, J., et al. (2021). Persistence of SARS CoV-2 S1 protein in CD16+ monocytes in post-acute sequelae of COVID-19 (PASC) up to 15 Months post-infection. Front. Immunol. *12*, 746021. https://doi.org/10.3389/fimmu.2021.746021.

84. Peluso, M.J., Lu, S., Tang, A.F., Durstenfeld, M.S., Ho, H.E., Goldberg, S.A., Forman, C.A., Munter, S.E., Hoh, R., Tai, V., et al. (2021). Markers of immune activation and inflammation in individuals with postacute sequelae of severe acute respiratory syndrome coronavirus 2 infection. J. Infect. Dis. *224*, 1839–1848. https://doi.org/10.1093/infdis/jiab490.

85. Theobald, S.J., Simonis, A., Georgomanolis, T., Kreer, C., Zehner, M., Eisfeld, H.S., Albert, M.C., Chhen, J., Motameny, S., Erger, F., et al. (2021). Long-lived macrophage reprogramming drives spike protein-mediated inflammasome activation in COVID-19. EMBO Mol. Med. *13*, e14150. https://doi.org/10.15252/emmm.202114150.

86. Bohnacker, S., Hartung, F., Henkel, F., Quaranta, A., Kolmert, J., Priller, A., Ud-Dean, M., Giglberger, J., Kugler, L.M., Pechtold, L., et al. (2022). Mild COVID-19 imprints a long-term inflammatory eicosanoid- and chemokine memory in monocyte-derived macrophages. Mucosal Immunol. *15*, 515–524. https://doi.org/10.1038/s41385-021-00482-8.

87. Robinson, P.C., Liew, D.F.L., Liew, J.W., Monaco, C., Richards, D., Shivakumar, S., Tanner, H.L., and Feldmann, M. (2020). The potential for repurposing anti-TNF as a therapy for the treatment of COVID-19. Med. (N Y) *1*, 90–102. https://doi.org/10.1016/j.medj.2020.11.005.

88. Investigators, T.R.-C., and Derde, L.P.G. (2021). Effectiveness of tocilizumab, sarilumab, and anakinra for critically ill patients with COVID-19 the REMAP-CAP COVID-19 immune modulation therapy domain randomized clinical trial. Preprint at medRxiv. https://doi.org/10.1101/2021.06.18.21259133.

89. Davidson, M., Menon, S., Chaimani, A., Evrenoglou, T., Ghosn, L., Graña, C., Henschke, N., Cogo, E., Villanueva, G., Ferrand, G., et al. (2022). Interleukin-1 blocking agents for treating COVID-19. Cochrane Database Syst. Rev. *1*, CD015308. https://doi.org/10.1002/14651858.CD015308.

90. Luo, P., Qiu, L., Liu, Y., Liu, X.L., Zheng, J.L., Xue, H.Y., Liu, W.H., Liu, D., and Li, J. (2020). Metformin treatment was associated with decreased mortality in COVID-19 patients with diabetes in a retrospective analysis. Am. J. Trop. Med. Hyg. *103*, 69–72. https://doi.org/10.4269/ajtmh.20-0375.

91. Li, J., Wei, Q., Li, W.X., McCowen, K.C., Xiong, W., Liu, J., Jiang, W., Marin, T., Thomas, R.L., He, M., et al. (2020). Metformin use in diabetes

prior to hospitalization: effects on mortality in covid-19. Endocr. Pract. *26*, 1166–1172. https://doi.org/10.4158/EP-2020-0466.

92. Bramante, C.T., Ingraham, N.E., Murray, T.A., Marmor, S., Hovertsen, S., Gronski, J., McNeil, C., Feng, R., Guzman, G., Abdelwahab, N., et al. (2021). Metformin and risk of mortality in patients hospitalised with COVID-19: a retrospective cohort analysis. Lancet Healthy Longev. *2*, e34–e41. https://doi.org/10.1016/S2666-7568(20)30033-7.

93. Xian, H., Liu, Y., Rundberg Nilsson, A., Gatchalian, R., Crother, T.R., Tourtellotte, W.G., Zhang, Y., Aleman-Muench, G.R., Lewis, G., Chen, W., et al. (2021). Metformin inhibition of mitochondrial ATP and DNA synthesis abrogates NLRP3 inflammasome activation and pulmonary inflammation. Immunity *54*, 1463–1477.e11. https://doi.org/10.1016/j.immuni.2021.05.004.

94. Aziz, N.A., Corman, V.M., Echterhoff, A.K.C., Müller, M.A., Richter, A., Schmandke, A., Schmidt, M.L., Schmidt, T.H., de Vries, F.M., Drosten, C., and Breteler, M.M.B. (2021). Seroprevalence and correlates of SARS-CoV-2 neutralizing antibodies from a population-based study in Bonn, Germany. Nat. Commun. *12*, 2117. https://doi.org/10.1038/s41467-021-22351-5.

95. Carfì, A., Bernabei, R., Landi, F., and for the Gemelli Against COVID-19 Post-Acute Care Study Group. (2020). Persistent symptoms in patients after acute COVID-19. JAMA *324*, 603. https://doi.org/10.1001/jama.2020.12603.

96. Arnold, D.T., Hamilton, F.W., Milne, A., Morley, A.J., Viner, J., Attwood, M., Noel, A., Gunning, S., Hatrick, J., Hamilton, S., et al. (2021). Patient outcomes after hospitalisation with COVID-19 and implications for follow-up: results from a prospective UK cohort. Thorax *76*, 399–401. https://doi.org/10.1136/thoraxjnl-2020-216086.

97. Huang, C., Huang, L., Wang, Y., Li, X., Ren, L., Gu, X., Kang, L., Guo, L., Liu, M., Zhou, X., et al. (2021). 6-month consequences of COVID-19 in patients discharged from hospital: a cohort study. Lancet *397*, 220–232. https://doi.org/10.1016/S0140-6736(20)32656-8.

98. Korsunsky, I., Millard, N., Fan, J., Slowikowski, K., Zhang, F., Wei, K., Baglaenko, Y., Brenner, M., Loh, P.R., and Raychaudhuri, S. (2019). Fast, sensitive and accurate integration of single-cell data with Harmony. Nat. Methods *16*, 1289–1296. https://doi.org/10.1038/s41592-019-0619-0.

STAR★METHODS

KEY RESOURCES TABLE

REAGENT or RESOURCE	SOURCE	IDENTIFIER
Biological samples		
Peripheral blood of individuals with prior COVID-19	This paper	N/A
Peripheral blood of healthy individuals	This paper	N/A
Plasma samples of individuals with acute COVID-19	Binder Lab	Schultheiß et al., 2020[37]
Critical commercial assays		
Anti-SARS-CoV-2-ELISA IgG	Euroimmun AG	Cat# EI 2606–9601 G
Anti-SARS-CoV-2-ELISA NCP	Euroimmun AG	Cat# EI 2606-9601-2 G
ANAScreen	Orgentec	Cat# ORG 538
Rheumatoid Factor Screen Orgentec	Orgentec	Cat# ORG 522S
Anti-Phospholipid Screen IgG/IgM Orgentec	Orgentec	Cat# ORG 529
LEGENDplex Human B Cell Panel (13-plex)	BioLegend	Cat# 740,527
LEGENDplex Human Anti-Virus Response Panel (13-plex)	BioLegend	Cat# 740,390
Software and algorithms		
R Studio version 4.1.1	RStudio, Boston, USA	https://rstudio.com/products/rstudio/
GraphPad Prism 8.3.1	GraphPad Software, La Jolla, CA, USA	https://www.graphpad.com/scientificsoftware/prism/
Seurat (v 4.0.3) R package	Satija Lab	https://satijalab.org/seurat/
Harmony R package	Raychaudhuri Lab	https://github.com/immunogenomics/harmony
Corrplot R package	N/A	https://github.com/taiyun/corrplot

RESOURCE AVAILABILITY

Lead contact

Further information and requests for resources and reagents should be directed to and will be fulfilled by the Lead Contact, Mascha Binder (mascha.binder@uk-halle.de).

Materials availability

This study did not generate new unique reagents.

Data and code availability

- All data reported in this paper will be shared by the lead contact upon request.
- This study did not generate new sequencing data or code.
- Any additional information required to reanalyze the data reported in this paper is available from the lead contact upon request.

EXPERIMENTAL MODEL AND SUBJECT DETAILS

COVID-19 module within the DigiHero cohort study (population-based cohort study for digital health research in Germany)

Until October 2021, 8,077 individuals took part in the digital cohort study DigiHero in the city of Halle (Saale), Germany. The recruitment was conducted in two waves and included mailed invitation to all 129,733 households in Halle as well as promotion via media. Of these, 919 individuals reported prior positive SARS-CoV-2 testing in their households and were thus invited – along with their

household members – to take part in the COVID-19 module of the study. Figure 1 provides a flow-chart of the COVID-19 module within the DigiHero study. Until ninth of October 2021, 318 individuals older than 14 years had been recruited to this module. All participants were interviewed with a questionnaire on the clinical course of COVID-19 and its sequelae as well as on vaccination status. As a validation cohort, another 333 DigiHero participants were included until 18th of February 2022. Overall, of 919 invited individuals, 651 individuals have taken part until the 18th of February 2022 resulting in a response rate of 71%.The study was approved by the institutional review board (approval numbers 2020-076) and conducted in accordance with the ethical principles stated by the Declaration of Helsinki. Informed written consent was obtained from all participants or legal representatives. The collected plasma samples were isolated by centrifugation of whole blood for 15 min at 2,000 × g, followed by centrifugation at 12,000 × g for 10 min and stored at - 80°C.

Biological samples and data from the biobank of the halle COVID cohort (HACO)

Plasma samples from acute bacterial pneumonia (n = 5), acute COVID-19 (n = 15 mild to moderate severity) and early post-acute COVID-19 (n = 49 mild to moderate severity) were recovered from the biobank of the HACO study that recruited participants from April to December 2020. Informed written consent was obtained and the study was approved by the institutional review board (approval number 2020-039) and conducted in accordance with the ethical principles stated by the Declaration of Helsinki. The collected plasma samples were isolated as described above.

METHOD DETAILS

SARS-CoV-2 antibody profiling

Antibodies against the S1 domain of the spike (S) protein and the nucleocapsid protein (NCP) of SARS-CoV-2 were determined by Anti-SARS-CoV-2-ELISA IgA/IgG and Anti-SARS-CoV-2-NCP-ELISA kits from Euroimmun (Lübeck, Germany). Readouts were performed at 450 nm using a Tecan Spectrophotometer SpectraFluor Plus (Tecan Group Ltd., Männedorf, Switzerland).

Cytokine and autoantibody profiling

Cytokine plasma levels were measured using the LEGENDplex Human B Cell Panel (13-plex) and the Human Anti-Virus Response Panel (13-plex) (BioLegend). For autoantibody screens, the Rheumatoid Factor (detects IgG, IgA and IgM RFs), ANA (detects SS-A 60, SS-A 52, SS-B, RNP-70, Sm, RNP/Sm, Scl-70, centromere B and Jo-1 IgGs) and Anti-Phospholipid IgG/IgM (detects cardiolipin, phosphatidylserine, phosphatidylinositol, phosphoglycerides und β2-glycoprotein one IgGs/IgMs) kits from Orgentec (Mainz, Germany) were used.

Single-cell transcriptome analyzes

Single-cell RNA sequencing data from previously published datasets was analyzed in R (v 4.1.1) using the package Seurat (v 4.0.3). For an integrated bronchoalveolar lavage fluid (BALF) dataset, we used seven patients with severe COVID-19 and four patients with bacterial pneumonia as disease control from Zhao et al.,[40] three patients with moderate COVID-19 from Liao et al.[42] and seven patients with severe COVID-19 from Wendisch et al[41] To analyze peripheral blood mononuclear cells (PBMCs) from COVID-19 patients we generated an integrated dataset encompassing all cells from patients with severe or mild COVID-19 as well as healthy individuals reported by Stephenson et al.[43] Schulte-Schrepping et al.[44] and Su et al[45] In addition, lung autopsy tissues from 16 deceased COVID-19 patients[39] was analyzed using the Broad Institute Single Cell portal https://singlecell.broadinstitute.org/single_cell/study/SCP1052. The BALF datasets were integrated as follows: First, normalization and detection of the top 2000 variable features was done individually for each dataset. Next, integration anchors were calculated using *FindIntegrationAnchors* and datasets were integrated with *IntegrateData* to one object. After rescaling of the integrated object, PCA and UMAP calculation were performed. Macrophage subsets were assigned according to the marker genes used by Zhao et al[40] The blood datasets were integrated using the package harmony[98] by creating an object from all concatenated count matrices with subsequent normalization, variable feature detection, scaling and PCA calculation. UMAPs were generated based on the first 20 dimensions of the harmony reduction. Since corresponding cell types from the single datasets clustered together after integration with very high accuracy, cellular identities of the integrated UMAP clusters were assigned according to the original publications. Cytokine response scores of gene sets were calculated using the Seurat function *AddModuleScore*. The IL-1β set encompassed *IL1R1*, *IL1R2*, *CASP1*, *NLRP1*, *NLRP3*, *TLR7*, *FOSB*, *NFKBIZ*, *NFKB1*, the IL-6 set *IL6R*, *IL6ST*, *CEBPD*, *NOTCH1*, *HES1*, *HES4*, *HEY1* and the TNF set *TNFRSF1A*, *TNFRSF1B*, *TNFSF10*, *TNFSF15*, *CLU*, *TNIP3*, *SIGLEC10*, *ENPP2*, *NKG7*, *TIMP1*, *CLEC5A* and *CCL7*.[48]

QUANTIFICATION AND STATISTICAL ANALYSIS

Barplots, plasma level heatmap and all statistical analyses were performed using GraphPad Prism 8.3.1 (GraphPad Software, La Jolla, CA, USA). Differences in plasma cytokine levels were studied by Welch's ANOVA and unpaired t-test with Welch's correction. Correlations were calculated using th R package corrplot. Linear regression analysis of plasma cytokine levels was performed using GraphPad and log10(x+1) transformed concentrations. Prism Ranges of p values are indicated with asterisks: *$p < 0.05$; **$p < 0.01$; ***$p < 0.001$; ****$p < 0.0001$.

Cell Reports Medicine, Volume *3*

Supplemental information

The IL-1β, IL-6, and TNF cytokine triad

is associated with post-acute sequelae of COVID-19

Christoph Schultheiß, Edith Willscher, Lisa Paschold, Cornelia Gottschick, Bianca Klee, Svenja-Sibylla Henkes, Lidia Bosurgi, Jochen Dutzmann, Daniel Sedding, Thomas Frese, Matthias Girndt, Jessica I. Höll, Michael Gekle, Rafael Mikolajczyk, and Mascha Binder

Table S1: Characteristics of participants in the DigiHero „COVID-19“ module, related to Figure 3 – validation cohort

	all participants	participants with confirmed prior COVID-19	Participants without prior COVID-19
Nb of participants	300	240	60
Sex			
Female	171 (57%)	143 (59.6%)	28 (46.7%)
Male	129 (43%)	97 (40.4%)	32 (53.3%)
Age (years)			
Median age	44	44.7	38
Range	14-87	14-86	14-87
Timing of COVID-19 infections			
Wave 1 (March 2020 -June 2020)		6 (2.5%)	
Wave 2 (July 2020 - Feb 2021)		153 (63.8%)	
Wave 3 (Mar 2021 - June 2021)		63 (26.3%)	
Wave 4 (from July 2021)		17 (7%)	
Not evaluated		1 (0.4%)	
Self rated acute infection severity			
asymptomatic		15 (6.3%)	
mild to moderate		208 (86.7%)	
severe		16 (6.6%)	
Not evaluated		1 (0.4%)	
Hospitalization		8 (3%)	
Intensive care unit		0	
Duration of symptoms			
not evaluated		18 (7.5%)	
0-4 weeks		75 (31.3%)	
4-12 weeks		29 (12%)	
>12 weeks		118 (49.2%)	
Nb of SARS-COV-2 vaccinations			
0		30 (12.5%)	7 (11.7%)
1		129 (53.75%)	6 (10%)
2		81 (33.75%)	47 (78.3%)
Type of SARS-CoV-2 vaccine			
not evaluated		13 (6.2%)	5 (9.4%)
mRNA vaccine*		122 (58.1%)	4 (7.55%)
mRNA vaccine + mRNA vaccine		53 (25.2%)	31 (58.5%)
vector vaccine*		7 (3.3%)	2 (3.8%)
vector vaccine + vector vaccine		5 (2.4%)	4 (7.55%)
vector vaccine + mRNA vaccine		10 (4.8%)	7 (13.2%)

* mRNA vaccines: Comirnaty (BioNTech / Pfizer) or Spikevax (Moderna Biotech),
vector vaccines: Vaxzevria (AstraZeneca) or Ad26.COV2-S (Johnson&Johnson)

Table S2: Duration of symptoms, related to Figure 3 – validation cohort

	< 4w		4-12w		>12w		ongoing	
Nb of participants with symptoms	217		143		97		65	
Fever	138	63.6%	7	4.9%	3	3%	1	1.5%
Lymph node swelling	56	25.8%	10	7%	5	5.2%	4	6.2%
Loss of taste/smell	167	77%	81	56.6%	37	38.1%	26	40%
Body aches	186	85.7%	59	41.3%	36	37.1%	29	44.6%
Fatigue	202	93.1%	105	73.4%	68	70.1%	49	75.4%
Sleeping disturbances	113	52.1%	61	42.66%	47	48.45%	38	58.5%
Lack of concentration	119	54.8%	81	56.6%	50	51.55%	40	61.5%
Anxiety	63	29%	34	23.8%	25	25.8%	21	32.3%
Depression	36	16.9%	34	23.8%	25	25.8%	22	33.8%
Headache	181	83.4%	54	37.8%	27	27.8%	20	30.8%
Coryza	168	77.4%	25	17.5%	16	16.5%	14	21.5%
Conjunctivitis	19	8.7%	5	3.5%	3	3.1%	2	3.1%
Otitis	42	19.3%	14	9.8%	10	10.3%	9	13.8%
Dyspnea	148	68.2%	87	60.8%	59	60.8%	44	67.7%
Sore throat	141	65%	24	16.8%	10	10.3%	9	13.8%
Cough	166	76.5%	51	35.7%	23	23.7%	17	26.2%
Angina	125	57.6%	54	37.8%	37	38.1%	29	44.6%
Heart complaints	46	21.2%	29	20.3%	25	25.8%	21	32.3%
Dizziness	109	50.2%	48	33.6%	29	29.9%	26	40%
Abdominal pain	48	22.1%	15	10.5%	11	11.3%	9	13.8%
Gastrointestinal compl	68	31.3%	23	16%	17	17.5%	15	23.1%
Nausea	41	18.9%	11	7.7%	8	8.3%	8	12.3%

w, weeks
